# The IMPROVE Guidelines (Ischaemia Models: Procedural Refinements Of in Vivo Experiments)

**DOI:** 10.1177/0271678X17709185

**Published:** 2017-08-11

**Authors:** Nathalie Percie du Sert, Alessio Alfieri, Stuart M Allan, Hilary VO Carswell, Graeme A Deuchar, Tracy D Farr, Paul Flecknell, Lindsay Gallagher, Claire L Gibson, Michael J Haley, Malcolm R Macleod, Barry W McColl, Christopher McCabe, Anna Morancho, Lawrence DF Moon, Michael J O’Neill, Isabel Pérez de Puig, Anna Planas, C Ian Ragan, Anna Rosell, Lisa A Roy, Kathryn O Ryder, Alba Simats, Emily S Sena, Brad A Sutherland, Mark D Tricklebank, Rebecca C Trueman, Lucy Whitfield, Raymond Wong, I Mhairi Macrae

**Affiliations:** 1 National Centre for the Replacement, Refinement and Reduction of Animals in Research (NC3Rs), London, UK; 2 The Roslin Institute and R(D)SVS, University of Edinburgh, Easter Bush, Midlothian, UK; 3 Faculty of Biology, Medicine and Health, University of Manchester, Manchester, UK; 4 Strathclyde Institute of Pharmacy and Biomedical Sciences (SIPBS), University of Strathclyde, Glasgow, UK; 5 Institute of Neuroscience and Psychology, College of Medical, Veterinary and Life Sciences, University of Glasgow/Arum Biosciences, Glasgow, UK; 6 School of Life Sciences, University of Nottingham Medical School, Nottingham, UK; 7 Comparative Biology Centre, Newcastle University, UK; 8 Department of Neuroscience, Psychology and Behaviour, University of Leicester, Leicester, UK; 9 Centre for Clinical Brain Sciences, University of Edinburgh, Edinburgh, UK; 10 Neurovascular Research Laboratory. Vall d'Hebron Research Institute, Universitat Autònoma de Barcelona; Barcelona, Spain; 11 Wolfson Centre for Age-Related Diseases, King's College London, London, UK; 12 Eli Lilly and Co., Windlesham, Surrey, UK; 13 Institut d’Investigacions Biomèdiques de Barcelona (IIBB), Consejo Superior de Investigaciones Científicas (CSIC), IDIBAPS, Barcelona, Spain; 14 NC3Rs Board, London, UK; 15 Home Office, London, UK; 16 Acute Stroke Programme, Radcliffe Department of Medicine, University of Oxford, Oxford, UK; 17 School of Medicine, Faculty of Health, University of Tasmania, Hobart, Australia; 18 Centre for Neuroimaging Sciences, Institute of Psychiatry Psychology and Neuroscience, King’s College London, London, UK; 19 Royal Veterinary College, London, UK

**Keywords:** 3Rs, animal welfare, guidelines, middle cerebral artery occlusion, stroke

## Abstract

Most in vivo models of ischaemic stroke target the middle cerebral artery and a spectrum of stroke severities, from mild to substantial, can be achieved. This review describes opportunities to improve the in vivo modelling of ischaemic stroke and animal welfare. It provides a number of recommendations to minimise the level of severity in the most common rodent models of middle cerebral artery occlusion, while sustaining or improving the scientific outcomes. The recommendations cover basic requirements pre-surgery, selecting the most appropriate anaesthetic and analgesic regimen, as well as intraoperative and post-operative care. The aim is to provide support for researchers and animal care staff to refine their procedures and practices, and implement small incremental changes to improve the welfare of the animals used and to answer the scientific question under investigation. All recommendations are recapitulated in a summary poster (see supplementary information).

## 1 Introduction

### 1.1 Background

Strokes are caused when there is an interruption in the blood supply to the brain, by either leaking (haemorrhagic stroke) or more commonly blocking (ischaemic stroke) of blood vessels supplying brain tissue. Despite significant reductions in stroke incidence and mortality in the last 25 years, over 100,000 people have a stroke in the UK every year which is fatal in 12% of patients in the first 30 days and represents a major cause of morbidity, with almost two-thirds of stroke survivors leaving hospital with a disability.^1^ Available treatments for ischaemic stroke are largely limited to mechanical or pharmacological (thrombolysis) strategies to re-open the blocked blood vessel and are suitable for only a small minority of patients. Other important strategies include preventing recurrent stroke, maximising the rehabilitation achieved and preventing or treating complications. There are at present no treatments, other than restoring cerebral blood flow (CBF), which protect the remaining brain substance and structures from the consequences of stroke (neuroprotection) or assist in repairing the brain (neuroplasticity).

Research using various approaches including animal models of stroke has led to a sophisticated understanding of the effects of ischaemia in the mammalian brain, and as far as we can tell, the majority of these pathological processes are largely shared across species including between rodents and humans. This identifies rodent models of ischaemic stroke (see O'Neill and Clemens^2,3^ and Howells et al.^4^ for overviews) as useful tools in the development and testing of novel treatments.

Many hundreds of compounds have been tested in experimental models of stroke^5^ and many of these appear to improve outcome. However, of almost 100 interventions which improved outcome in animal stroke models and which were tested in clinical trials, only one (re-opening the blocked blood vessel with recombinant tissue plasminogen activator (rtPA)) improved outcome in humans.^5^

In some cases the reasons for this failure of translation are readily apparent: gavestinel (an antagonist of glycine at the N-methyl-D-aspartic acid (NMDA) receptor) improves outcome in animals, but has very low penetration to the human brain and cerebrospinal fluid (CSF); tirilazad was effective in animals when given a median of 10 min after stroke onset, but not in human clinical trials when the delay to treatment was more than 3 h for three quarters of the patients included in the study. For others, systematic review of the supporting animal literature suggests that the apparent efficacy observed in those studies may have been due to suboptimal experimental design.^6^ For instance, studies which do not report strategies such as randomisation or blinding to reduce the risk of bias tend to give inflated estimates of drug effect; and in the past only a minority of stroke studies reported using any such strategies. Finally, it may be that the animal literature apparently supporting efficacy represents only a subset of the experiments performed, the others being resolutely neutral or negative, but not published. This problem of publication bias has been estimated to lead to an overstatement of efficacy of around 30%^7^; and publication bias magnifies the effects of inadequate sample size (and therefore statistical power), where it is only the studies which (by chance) show extreme effects which reach the published literature.

These problems are by no means limited to the modelling of stroke, but because they were first identified here, the in vivo stroke community has been well placed to take the lead in addressing them. This was manifest in the Stroke Therapy Academic Industry Roundtable (STAIR) criteria,^8,9^ model-specific good laboratory practice guidelines^10^ and changed editorial policies at both the *Journal of Cerebral Blood Flow and Metabolism* and *Stroke*. In a recent assessment of reporting standards for all in vivo research from leading UK institutions, only one publication met all four reporting criteria, and this publication described in vivo stroke research.^11^

Despite this progress, there are opportunities to further improve the in vivo modelling of ischaemic stroke. This will not only increase the reliability and economic productivity of research but will also improve the skill base of the scientific workforce and, by maximising the value of the information gained from animal research, will provide a firmer ethical basis for such work. Against this background we consider here issues of animal welfare, and provide a number of recommendations for strategies to minimise pain and distress in these models, and thereby also improve scientific outcomes.

### 1.2 Working group

The National Centre for the Replacement, Refinement and Reduction of Animals in Research (NC3Rs) is a scientific organisation established by the United Kingdom (UK) Government in 2004 to lead the discovery and application of new technologies and approaches to replace, reduce and refine the use of animals for scientific purposes. In 2014 the NC3Rs convened an expert Working Group with the following terms of reference:

1. To review the most commonly used rodent (mouse and rat) models of stroke.
2. To identify the animal welfare issues.
3. To recommend opportunities for refinement.
4. To publish the deliberations of the Working Group and promote its recommendations within the international stroke research community.

The overall aim was to provide recommendations which might reduce the level of severity experienced by animals in the most common models of middle cerebral artery (MCA) occlusion whilst at the same time sustaining or increasing the value of the science. The Working Group consisted of UK experts from academia, the pharmaceutical industry and the UK Home Office (the government body with responsibility for animal research). In addition, several members of the group, identified in the author list, were participants in the Framework 7 Multi-PART project (http://www.dcn.ed.ac.uk/multipart/), which aims to overcome the problem of poor translation of preclinical stroke research by providing a platform for international, multi-centre preclinical trials to test new therapies, thus applying the same standards and methodology used in clinical research. The information and recommendations of the Working Group have drawn in part on the standard operating procedures proposed by the Multi-PART team. In addition, some of the authors participated in a workshop to exchange best practice and identify refinements for specific models.

### 1.3 Considerations of the choice of model

The majority of rodent ischaemic stroke models target the MCA, with either transient or permanent occlusion. The main methods of MCA occlusion (MCAO) are: (1) mechanical – e.g. blocking the origin of the MCA intraluminally with a filament, using clips and/or sutures to tie off the artery or applying compression to stop the flow through the artery; (2) electrocoagulation – coagulating the blood and destroying the structure of the artery using fine diathermy forceps; (3) pharmacological – e.g. applying vasoconstrictor substances such as endothelin-1 directly onto the artery or injecting it into neighbouring tissue to induce prolonged, local ischaemia; (4) thrombo/embolic – introducing a pre-formed blood clot to block the MCA, or its distal branches, inducing local thrombosis with thrombin injections or a combination of Rose Bengal and a laser to form the blood clot.^4^

While these four main methods of inducing stroke are those considered in this paper, modifications and refinements of these models extend the range even further. For example, subcortical structures can be spared and a pure cortical infarct produced by occluding a more distal portion of the MCA or its branches (methods 1, 2 & 4); severity of ischaemia can be increased by simultaneous uni- or bilateral common carotid artery (CCA) occlusion or controlled hypotension (methods 1, 2, 3 & 4). Duration of ischaemia can be controlled (method 1) and severity/duration influenced by adjusting the concentration or volume of endothelin-1 (method 3) or the characteristics of the laser in the Rose Bengal model (method 4). Therefore, a spectrum of stroke severities, from mild to substantial, can be achieved using the various models.

Recommendations on which models are most appropriate to use have been the subject of recent extensive reviews^12,13^ and are not covered here. Similarly, good practice in both experimental design^10^ and reporting^14^ is also outside the remit of this paper.

As a guiding principle when designing stroke studies, the model used should deliver the minimum severity and duration of ischaemic insult required to answer the scientific question under investigation or the hypothesis being tested. In addition, there are two aspects of stroke severity to consider: that arising in the course of inducing the model itself, and the severity of the individual animal outcome. In general the severity and duration of the ischaemic insult are adjusted to achieve a measureable and reproducible primary outcome measure. As is the case in stroke patients, however, there is often a large heterogeneity in outcome including morbidity and mortality within a group of animals exposed to a standard ischaemic insult. Outcome data are more variable in models of transient MCA occlusion than in permanent occlusion as transient models can cause vascular endothelial damage and secondary reperfusion events may add to the variability. The aim should be to reduce unwanted variability as much as possible to improve signal to noise ratio and increase the power of the experiments. Factors such as sex, strain, age and environment also affect the outcome measure which implies that modification of the environment to improve welfare will have an impact on the outcome, particularly on the extent of survival and recovery, as described below.

The structure of the present paper is organised as a timeline, and different sections follow the order these things would be encountered during the course of the experiment, from acclimatisation of the animals before surgery and choice of anaesthetic regimen through to the surgery itself and post-operative care. These considerations apply to all rodent ischaemic stroke models, whereas the last section covers refinements for specific models. The recommendations are highlighted in bold at the end of each section. They are also recapitulated in a summary poster, which also includes the humane endpoints and the traffic light system to monitor the animals after stroke (see supplementary information; the poster would be best printed off in size A3).

## 2 Basic requirements pre- and post-stroke

### 2.1 Acclimatisation pre-stroke

Animals should be acclimatised to new facilities for at least seven days prior to experimental work commencing and visual inspection of animals to check baseline normal health status should be undertaken over this time. Assessing the behaviour and appearance of the animal (e.g. grooming and coat condition) as well as body weight not only allows a check of its wellbeing, but also allows any deviation from normal to be recognised.^15^ Animals should be weighed daily, for at least three days before the surgery, as this allows a normal growth curve to be constructed. This enables any fall in weight after the procedure and the recovery of weight to be assessed appropriately.

There is evidence that rodent behaviour, physiology and sleep patterns are affected by transport.^16–18^ Physiologically, a number of factors in rodents are affected, even by routine in-house transport, and can lead to increased levels of plasma corticosterone,^19^ decreased immune activity^19^ and decreased body weight despite continued access to food and water.^20^ These effects can take two to four days to normalise.^21,22^ However, normalisation of behaviour takes even longer. Rearing, climbing, grooming, feeding and sexual behaviours of mice change significantly immediately after transportation and take more than four days to normalise.^23^ Of note is that transport between continents (as can be the case with transgenic mice) may induce an extended light/dark time which causes circadian rhythm to take more than two weeks to resynchronise.^24^ In addition, other environmental disturbances including husbandry, common experimental procedures such as handling, injections and even noise/vibrations due to building/infrastructure work can alter physiology (increased heart rate and mean arterial blood pressure) and behaviour (for review Turner et al.^25^). It is important that there is stability in the day-to-day influences on the animals so that variability is reduced.

Many species of animals live in hierarchies that involve dominance and submission. Re-housing animals in new groups may be a stressful experience if animals are not compatible. It may disrupt social relationships, cause aggression and should be avoided unless absolutely essential.^26,27^ Accordingly, it is recommended that research groups avoid mixing their animals when they arrive in the unit and animals should keep the same cage mates throughout the experiment and should only be randomised to treatment.

Before the experimental procedures start, rodents should be acclimatised to handling by the same experimenter; this can be done daily during the acclimatisation period. Evidence shows that regular handling before behavioural testing has an anxiolytic effect in both rats^28^ and mice.^29^ Handling methods such as cupping mice with open hands or using tunnels are preferable to the traditional approach of picking it up by the tail, which has been shown to be aversive and to induce high levels of anxiety in several strains of mice.^29,30^ For rats, there are no data on tunnels and cupping is not possible, but rats should be handled by grasping around the shoulders, not by the tail. Reducing anxiety of handling will ensure that the stress response does not confound the scientific outcome.

1. **Rodents ordered from an outside supplier should be delivered at least seven days before the procedure to allow acclimatisation to the new environment.**
2. **Animals should be acclimatised in harmonious groups before the start of the experiment. Re-housing animals in new groups should be avoided.**
3. **Animals should be acclimatised to handling and should not be handled by the tail. Tunnel and cup handling should be used for mice; rats should be handled by grasping around the shoulders.**
4. **Animals should be weighed daily for at least three days before surgery.**

### 2.2 Cage enrichment

The Guide for the Care and Use of Laboratory Animals^31^ has defined environmental enrichment as the provision of structures and/or resources that improve sensory, motor and psychological wellbeing of the animal through physical exercise, manipulative activities and cognitive challenges according to species-specific behaviours. The provision of environmental enrichment is a basic rodent housing and husbandry requirement to improve overall wellbeing^32^ (https://www.nc3rs.org.uk/our-resources/housing-and-husbandry/rodents).

As described below, many research groups have now shown the benefits of ‘enriched environments’ relative to ‘standard housing’ in both naïve and lesioned laboratory animals. However, it is important to remember that ‘enriched environments’ always fall short of the natural environment that animals experience in the wild (e.g. free access to mates, unrestricted three-dimensional complex space to roam, hunting for food rather than passive ‘chow’). We must recognise that laboratory animals always live in impoverished environments relative to animals living in the wild. Thus any conclusion such as ‘environmental enrichment increases neurogenesis’ might arguably be better phrased as ‘standard (impoverished) housing causes a deficit in neurogenesis’. Environmental enrichment can be considered as a form of refinement based on the animals’ needs that improves wellbeing. Therefore it is recommended that the impact on scientific method, outcome and statistical power are evaluated to ensure the animal model remains intact and to ensure valid conclusions can be drawn.^33^ Environmental enrichment in laboratory rodents has the potential to increase variability in the data.^34^ However it does not increase the risk of obtaining conflicting data in replicate studies.^35^ In addition, the level of enrichment must be considered carefully given mounting evidence for neurorestorative effects of ‘super-enrichment’ as described below.

In Europe, minimum requirements for the level of enrichment for standard housing conditions are set by the EU Directive 2010/63,^27^ which stipulates that animals should be provided with ‘space of sufficient complexity to allow expression of a wide range of normal behaviour’. Minimum cage sizes are prescribed for each species depending on the weight of the animals. For example, 35 g mice require a minimum enclosure size and height of 330 cm^2^ and 12 cm, respectively, with at least 100 cm^2^ of floor area per mouse, while 350 g rats require a minimum enclosure size and height of 800 cm^2^ and 18 cm (note that this height is not sufficient for adult rats to rear up; in the UK, the minimum height is 20 cm^36^), respectively, with at least 350 cm^2^ of floor area per rat.^27^ As well as cage substrate, bedding, nesting material and refuges are considered essential for laboratory rodents and should only be withheld for veterinary or welfare reasons.^37^ It is important that cages contain sufficient nesting materials prior to and after stroke. Laboratory mice are typically housed well below their lower critical temperature, which is around 30℃.^38^ Deep, thick nests can help control hypothermia and allow thermoregulation, thus reducing the impact that thermal stress could have on the scientific outcome.^38^ It is important to provide plenty of nesting materials at least one day before surgery so that the animals can assemble their nests as they are likely to do this less well after stroke. However, the bedding must be clean after surgery and it should be renewed if soiled so animals with wounds are not lying on soiled bedding. Some research groups provide animals with cages containing plastic housing and opportunities for exercise. For example, the company Bio-Serv (https://www.bio-serv.com) sells ‘Igloos’ that mice nest in. These Igloos may be purchased with a simple wheel (an off-horizontal spinning disk) that mice spend a great deal of time running upon. These are autoclavable and relatively inexpensive. Chew bars (e.g. aspen sticks) should be placed into the cages of animals; this allows expression of normal gnawing behaviour. It may also help prevent incisor tooth overgrowth, which is a particular concern in old animals or animals on a soft diet.

#### 2.2.1 Super-enriched environments

A higher level of enrichment has been termed ‘super-enriched’ and provides more complex, multisensory stimulation through use of, for example, larger multilevel cages, access to novel and varied toys and equipment for voluntary exercise (ladders, running wheels), music, odours and hidden treats.^39^ Whilst evidence exists for lack of effects of super-enriched environments on clinical pathology and cardiovascular parameters,^40^ it should be noted that using super-enriched environments, over and above standard cage enrichment, has been shown by a meta-analysis of rodent studies to improve functional outcome in models of stroke.^41^ Using these sorts of super-enriched environments should therefore be carefully considered, not only as an improvement in animal welfare, but also because of possible enhancement of the effects of neurorestorative strategies (for reviews see Mering and Jolkkonen^39^ and Nithianantharajah and Hannan).^42^ The effects of super-enriched environments can be dependent on the quantity of environmental enrichment,^43^ they may possibly be independent of infarct size^41^ and they may possibly involve lesion-induced increases in cell proliferation in the subventricular zone.^44^ In addition, if a key goal of the study is to evaluate functional recovery (behavioural read-outs) these enrichments may also alter the baseline rate of recovery post-stroke. Therefore additional enrichment should be considered carefully^45^ and it is important that the quantity and constituents of environmental enrichment are clearly specified when reporting studies in the literature.

#### 2.2.2 Special issues relating to animal models of stroke

Animal models of stroke often deliberately aim to model co-morbidities found in stroke patients, including sedentary lifestyles, high fat diets, obesity and diabetes. Accordingly, stroke researchers may assert that there may be some face validity in restricting aspects of cage enrichment. For example, it may be counter-productive to provide mice with ad libitum access to running wheels before or after stroke unless lean animals and active rehabilitation are desired. Such decisions should be overt and reported accordingly.

5. **Cage substrate, nesting material and shelter are basic welfare needs for rodents and should be provided. Tunnels, wheels and chewing sticks are simple, cost-effective ways to improve enrichment.**
6. **Additional ‘super-enrichment’ should be considered carefully, as it may have neurorestorative effects. Enrichment should be reported in publications specifically.**

### 2.3 Bedding material post-surgery

Following surgery, the bedding material must be chosen so as to avoid complications with feeding and drinking and not interfere with wound healing. In practice, there are a number of possibilities, none of which are perfect or obviate the need for close monitoring. Several researchers avoid the use of standard cage substrate/bedding as sawdust or wood chippings/shavings can get caught in the airway of rodents, and long strands of paper (that resemble those produced by a shredder of confidential documents) are not recommended as limbs of animals with impaired mobility or sensation can get caught in the strands. Others prefer to use absorbent, disposable tray liners or infant bed mats such as those made by the nappy (diaper) company, Huggies. These ‘DryNites’ come with adhesive strips that can be folded to stick onto the floor of a rat cage. Some groups place these into the cage the day before surgery to enable acclimatisation by the animals, although rodents may shred the liners. Liners allow monitoring of normal body function and can indicate if the animals are urinating and defecating. Other groups prefer the non-fibrous, dust-free pelleted paper product, 3Rs LAB bedding (http://3rsbedding.com) which has the advantage of being warm, suitable for nesting by both rats and mice and requiring infrequent changes because of its high absorbency. The choice of product should be made in consultation with the local vet or animal care staff.

7. **In consultation with the veterinary and animal care staff, consideration should be given to the bedding materials and any new material should be introduced prior to surgery to acclimatise the animals.**

### 2.4 Social housing post-stroke

Social grouping is an important component of environmental enrichment by allowing species-typical characteristics such as fighting, playing or sleeping together.^46^ Animals deprived of the possibility to perform species-specific behaviour may show behavioural disorders or other indicators of chronic stress, including pathological changes. As outlined by the EU Directive 2010/63 and in section 2.1 ‘Acclimatisation pre-stroke’, we recommend housing animals as soon as is possible in the same groups after stroke as they were housed in before stroke.

Ample evidence shows that recovery from surgery is improved in mice housed in groups.^47^ Social isolation following stroke increases infarct volumes and decreases brain-derived neurotrophic factor (BDNF) levels in mice^48^ and alters the neuroinflammatory response to stroke.^49^ The protective effects of social housing after stroke do not appear to be mediated by passive transfer of body heat but physical contact does appear to be necessary.^49^ Concern that cage mates will remove sutures from each other’s surgical wound is rarely a significant issue, provided wounds are closed competently. Use of appropriate gauge and type of suture material, and use of a subcuticular closure technique can eliminate these concerns. Tissue glue can also be used in addition to a subcuticular closure.

Animals with small strokes (e.g. small subcortical infarcts) can be group-housed immediately on recovery from general anaesthesia but for larger strokes, where the animals’ behaviours are significantly affected, it may be advisable to house individually in the immediate post-surgical period to provide additional care, and allow some degree of recovery and wound healing before group-housing. Even with a delay of as much as 72 h post-stroke, pair housing still leads to enhanced functional recovery.^50^

While social housing of mice with a healthy (non-stroke) partner immediately after stroke reduces mortality compared to housing with a stroke partner,^50^ there are implications for long-term social housing, and careful experimental design will be required depending on the nature of the partner animals which may be either sham-operated (but non-stroke) or naive (non-operated). If the experiment involves sham-operated and stroke groups, co-housing the animals is both a welfare benefit and good scientific practice. This should be built into the randomisation protocol to ensure that each cage will contain sham-operated and stroke animals.^51^ Similarly, to rule out cage effects, animals within each cage should be randomised to different treatment groups. Alternatively, if the animals’ behaviour is too significantly affected by the stroke to be housed with sham-operated or naïve animals, the same surgery may be performed on all animals in one cage. This however has implications for the design and analysis of the experiment, and in such cases, the experimental unit would be the cage rather than the individual animal.

We recommend a pragmatic strategy for monitoring group-housed animals (see section 6.1 ‘Monitoring of animals’). If there is a suspicion or evidence that an animal is behaving aggressively towards its cage mates, then it may need to be removed until all other animals in the cage have recovered adequately. It is plausible that a sham-operated animal housed with impaired stroke animals may seek to change the hierarchy by asserting dominance where previously it was submissive. Video monitoring with an appropriate web cam can be used to detect such behaviours during ‘lights out’ phase.

The issue of food and water post-stroke is dealt with in the next section. Group-housing does prevent the measurement of food and water intake (unless specialised equipment is used) but the benefits of group-housing are thought to be more important.^15^ One way to ensure equal access to food is to provide more food bowls than animals in each cage. Scattering food pellets and several packs of recovery gel on the cage floor also makes access to food more equitable for group-housed animals.

8. **After stroke, animals should be returned to the same group of animals they were with before surgery as soon as they are sufficiently recovered.**
9. **The randomisation protocol should ensure that each cage contains sham-operated and stroke animals, and/or animals allocated to different treatments.**

### 2.5 Acclimatisation to post-stroke supplementary diet

Mice and rats are known to be neophobic and therefore resistant to eating or drinking anything they do not recognise.^52,53^ It is crucial that they are habituated to any novel food or drink before the start of the experiment and that surgery is not undertaken until animals are acclimatised to it (see section 6.3 ‘Supplementary fluids and diet’ on supplementary fluid and diet post-stroke) to ensure that feeding and drinking activities after the surgery are not reduced because of neophobia.

Feeding behaviour in rats and mice follows a diurnal cycle, with the majority of food consumed at night or during the dark phase.^17,18^ To respect the circadian rhythm of the animals, new food should be put in the cage before the active phase when animals will be feeding.

10. **Animals should have access to their post-stroke diet prior to surgery and surgery should not be undertaken until they reliably consume the diet.**

### 2.6 Food restriction pre-surgery

Withdrawal of food before general anaesthesia is generally not necessary in rodents as they lack the emetic reflex^54^ and there is no risk that they will vomit during surgery. Overnight fasting can actually be harmful in rodents because of their high metabolic rate. It leads to changes in pharmacokinetics, which may significantly impact the response to the drug under study and can cause stress, aggressive behaviour and reduction in body weight, temperature and plasma glucose levels.^55^ Even withdrawal of food for 6 h can lead to weight loss and depletion of liver glycogen.^56^ Access to water should never be restricted prior to general anaesthesia.^57^

Food restriction can be justified for experiments specifically designed to study hyperglycaemia as, without fasting or food restriction, blood glucose can be high following the induction of anaesthesia^58,59^ and confound the outcome of the experiment. Rather than fasting animals overnight, it is preferable to provide a limited amount of food so that it runs out in the evening and the animals have an empty stomach the next morning. Prior to surgery, food may also be restricted for other scientific reasons, including behavioural testing that uses a food reward. Examples would include skilled reaching in the staircase test^60,61^ and various operant/lever-based tasks or touchscreen-based tasks. However, in all these cases it is advised that animals are pre-trained to a specified criterion and then returned to food ad libitum for at least one dark phase and an assessment made of their clinical condition (e.g. body weight and body condition score) prior to surgery.

11. **Rodents should not be routinely fasted before surgery, unless there is a scientific reason; any restriction should be reported specifically in publications.**
12. **After any food restriction for training purposes, sufficient time should be left to re-establish normal feeding patterns before surgery.**

### 2.7 Factors influencing host microbiota composition

The influence of microbiota composition and dysregulation on a wide array of physiological and pathological processes involved in health and disease is increasingly recognised.^62^ In the context of CNS development and function, the microbiota has been shown to exert important influences through multiple mechanisms including direct neural and humoural communication and via regulation of the endocrine, metabolic and immune systems, all of which themselves have important effects on CNS homeostasis and pathology, including stroke.^63,64^ A number of recent studies have shown how experimental stroke in rodents modifies the gut microbiota composition, in part related to alterations in gut permeability and motility.^65–68^ In addition, several studies have shown that controlled alterations to microbiota composition prior to stroke (e.g. in germ-free conditions or induced by antibiotic treatment) influence pathological and functional stroke outcome measures including through immunological mechanisms).^66,67,69^ Many factors pertinent to rodent stroke studies influence microbiota composition including animal facility hygiene status, species and strain, housing arrangements, diet and handling techniques.^64^ It is therefore important to aim for consistency in inter-animal and inter-study housing, feeding and handling of animals (and cages including any associated materials) such that potential inadvertent and uncontrolled influences on microbiota are avoided. It is also important to consider possible roles of microbiota alterations and downstream mechanisms (e.g. immune perturbation) in studies where environmental conditions are deliberately altered to test the effects on outcome (e.g. co-housing versus individual housing of animals after stroke).

13. **Consistency of inter-animal housing, feeding and handling practices before and after stroke should be ensured.**

### 2.8 Specific requirements for aged animals and those with co-morbidities

A major shortcoming of experimental stroke studies is that they tend to ignore confounding factors, or co-morbidities, which are known to impact upon stroke outcome. Such confounding factors include increased age, hypertension, diabetes, obesity, infection, inflammation and atherosclerosis.^70,71^ Age is considered to be the most important independent risk factor for stroke with stroke rates, in humans, doubling every decade after the age of 55.^72^ In addition, age is a significant predictor of outcome, independent of stroke severity, aetiology, thrombolysis, gender and other vascular risk factors.^73^

Very few experimental studies exist in the literature comparing outcomes after stroke in both middle-aged and older animals and these tend to report inconsistent results. Different studies have shown that, in comparison to young males, aged males may have larger infarcts, smaller infarcts or equivalent infarct size. In spite of disagreements on the effects of aging on infarct volume, invariably significantly higher mortality rates and more severe neurological impairments are found in older animals, consistent with clinical data.^74–78^ Such detrimental effects, in terms of function and animal welfare, are a direct consequence of the ageing process per se rather than the amount of ischemic damage generated. If the infarct volume is reduced in aged females to that seen in young females by hormone supplementation, the poorer functional ability and increased mortality remain in the aged females.^74^ Therefore, the stroke model may need to be adjusted and more intensive post-stroke care required in aged animal studies to minimise morbidity and mortality. This also applies to animal models expressing co-morbidities associated with stroke such as hypertension, diabetes, hyperlipidaemia and metabolic disease. In addition, if aged animals, or those with other co-morbidities, are being included in studies then this needs to be considered during the experimental design stage as mortality rates may differ compared to studies using young, healthy animals.

In aged animals it is important to monitor body weight and general welfare of the animals whilst they are being housed (i.e. even before they have undergone stroke surgery). A programme of regular monitoring should be in place to detect clinical signs that may indicate a problem (see https://www.sharmuk.org/join-sharm-community/welfare for further information). If aged animals begin to experience weight loss this may be caused by overgrown teeth which affect their ability to eat. It is considered good practice to provide aged animals with chew blocks and to regularly check their teeth. If overgrown teeth are identified they can be trimmed, under brief general anaesthesia, using a rotary dental disk cutter (for video see Wayman et al.^79^). If overgrown teeth are identified then it is likely that they will require fairly frequent treatment (e.g. every two to four weeks) as overgrowth commonly recurs probably due to misaligned upper and lower incisors. If aged animals start to exhibit weight loss or other changes in condition (e.g. piloerection) that is not a result of recent surgery or overgrown teeth then it is necessary to seek veterinary advice. If appropriate intervention cannot restore the lost body weight or body condition then it is recommended that animals are humanely killed (see section 6.1 ‘Monitoring of animals’ on monitoring of animals post-stroke). Magnetic resonance imaging (MRI) shows that elderly Lister Hooded and Long Evans rats displaying these symptoms often have pituitary tumours (Lawrence Moon, personal communication). Pituitary adenomas are also a common occurrence in aged Sprague Dawley rats.^80^ If present, they are inoperable and animals should be humanely killed. Presence of co-incident disease or signs suggestive of it, which is not the co-morbidity under test, will probably alter scientific results. Use of such animals, or their removal from the study should be reported in publications.

14. **Aged animals and those with co-morbidities should receive extra monitoring.**
15. **Teeth should be checked regularly, especially if the animal is on soft food diet. Animals should be provided with chew sticks to grind teeth.**

## 3 Anaesthesia and analgesia

### 3.1 General considerations

Knowledge of the general physiology of the animal and its responsiveness to pharmacological agents, as well as consideration of the duration of surgery, are essential for selecting the most appropriate anaesthesia. Factors such as age, strain, sex or body weight are known to influence the pharmacokinetics and metabolism of anaesthetic agents.^57^ Consideration should be given to animal characteristics and particularly so with animals harbouring co-morbidities or in very young or old animals. It may be prudent to optimise the anaesthetic regimen in pilot studies where anaesthesia response might be difficult to predict. Many anaesthetic agents require metabolism by the liver and/or renal excretion, so recovery from anaesthesia may be compromised in old or co-morbid animals. A notable exception is isoflurane, which is 99% exhaled, and may be a good choice for these animals. There are also significant strain differences in response to anaesthesia, particularly with injectable agents,^57,81,82^ so caution is advised when extrapolating doses between different strains of the same species.

Anaesthesia is required for induction of stroke with almost all of the currently used models. Most studies involve recovery of the animal following stroke induction, and so consideration will also need to be given to post-operative care and analgesia. Anaesthesia may need to be repeated following lesion induction, in order to monitor the progression of ischaemic damage and recovery, for example by use of functional MRI (fMRI). Anaesthesia can have both direct and indirect effects on the extent of ischaemic damage induced, and other factors in the perioperative period can also introduce variability to the model. Many of these factors were identified in the STAIR guidelines, with the recommendation that they should be controlled as part of good study design.^8^ However a recent review of papers reporting stroke induction in rodents indicates that the majority of studies do not report use of measures to minimise the effects of anaesthesia, such as the use of intubation and controlled ventilation, or maintenance of adequate oxygenation.^83^

The choice of anaesthetic agent should reflect both what is best for the welfare/physiology of the animal and also the requirements of the experiment. Some of the commonly used and recommended anaesthetics are listed in Table 1 with some comments on their use; however the choice of anaesthetic agent should be based on a careful review of the relevant literature, particularly when using stroke models to assess potential pharmacological interventions.

**Table 1.** Commonly used general anaesthetics.

	Dose (mouse)	Dose (rat)	Comments
**Gaseous**			
Isoflurane	Allow animal to breathe 100% oxygen for 1 min before induction. Induce at 5% isoflurane until anaesthetised, then reduce quickly to maintenance level (usually 1.5–2%)		Isoflurane is more potent than sevoflurane but the latter has faster induction and recovery (Fish et al., 2011)^92^
Sevoflurane	As above, with induction concentrations of 8% and maintenance of 2.5–3.5%		
Halothane	As above, with induction concentrations of 4% and maintenance of 1.25–1.75%		No longer commercially available in many countries
Ether	Not recommended on health and safety or humane grounds		Causes mucosal irritation and forms explosive mixtures with both air and oxygen
**Injectable**			
Propofol	26 mg/kg i.v. as bolus to induce. Maintain at 20–25 mg/kg/h infusion	10 mg/kg i.v. to induce. Maintain at 20–25 mg/kg/h infusion	Propofol i.v. gives consistent and stable anaesthesia, but venous access is more difficult in mouse
Ketamine/xylazine	80–100 mg/kg ketamine + 10 mg/kg xylazine i.p.	75–100 mg/kg ketamine + 10 mg/kg xylazine i.p.	Causes hyperglycaemia and peripheral vasoconstriction. Anaesthesia can be partly reversed with atipamezole s.c.
Ketamine/ medetomidine	75 mg/kg ketamine + 1.0 mg/kg medetomidine s.c. or i.p.	75 mg/kg ketamine + 0.5 mg/kg medetomidine s.c. or i.p.	Similar to ketamine/xylazine, anaesthesia can be partly reversed with atipamezole s.c.
Tribromoethanol (Avertin)	Not recommended for recovery surgery		Avertin can cause peritonitis
Medetomidine/ fentanyl/midazolam	0.5 mg/kg + 50 µg/kg + 5 mg/kg s.c.	150 µg/kg + 5 µg/kg + 2 mg/kg s.c.	Similar to ketamine/xylazine but anaesthesia can be completely reversed with atipamezole, naloxone and flumazenil s.c.
Medetomidine (following induction with 5% isoflurane then 1% for maintenance)	–	50 µg/kg bolus dose followed 15 min later by 100 µg/kg/hour s.c. infusion	This protocol can be used for fMRI experiments, in which isoflurane may cause a loss of signal. The use of medetomidine allows the concentration of isoflurane to be reduced. Bradycardia is a normal side effect and the bolus injection of medetomidine induces a pronounced drop in blood pressure. Medetomidine can be reversed with atipamezole 100 µg/kg s.c.
Pentobarbital	Not recommended		Depth of anaesthesia cannot be controlled safely unless administered intravenously. No longer available as a commercial anaesthetic product in many countries.

The data in this Table are from the text of section 3.1 ‘General considerations’ or from additional references.^88,176,177^

In experiments including sham-operated animals it is imperative that they are exposed to exactly the same duration of anaesthesia as MCA occlusion-operated animals accounting for the pre-occlusion surgery, occlusion and post-occlusion wound closure in order to separate out stroke-specific effects. Of note, previous studies have documented the effects of even brief periods of anaesthesia in comparison to non-anaesthetised animals on stroke-related outcome measures.^84^ Guidelines on good research reporting practice suggest that these details are important to allow accurate interpretation and reproducibility of studies.^14^

### 3.2 Direct effects of anaesthetic agents

A range of anaesthetic agents are available for use in rodents, and most, if not all, have the potential to influence outcomes of stroke models, either directly or indirectly. Both inhalational agents and injectable agents can be used. Because of practical considerations, most injectable anaesthetics are administered by the intraperitoneal route to small rodents, an approach that inevitably introduces additional variation because of the relatively high incidence of extra-peritoneal administration of some of the injectate.^85^ Intravenous administration of anaesthetics is relatively straightforward in both rats and mice, but maintenance of anaesthesia by continuous infusion is technically more difficult in mice. In rats, placement of a catheter percutaneously in the tail vein enables total intravenous anaesthesia, with excellent control of anaesthetic dose and depth of anaesthesia.^86,87^ It is, however, often easier to use inhaled anaesthetics, as the onset and recovery from anaesthesia is rapid and the length and duration of anaesthesia more controllable.^88^

Anaesthetic agents can have direct effects on the size of the ischaemic lesion and its progression, either through neuroprotective activity, by effects on neurotransmitters and receptor systems, or by induction of hyperglycaemia. Modification of neuroprotective pathways by general anaesthetics has been widely described. These effects should be taken into account in studies testing efficacy of a putative neuroprotective compound. Nonetheless, since anaesthesia is a prerequisite in animal stroke modelling it should be recognised that any ‘protective’ effects are only relative to an alternative agent. Isoflurane and sevoflurane improve neurological outcome compared with fentanyl in rat models of ischaemia,^89,90^ possibly via a reduction of sympathetic activity.^90^ Isoflurane was also shown to preserve spatial memory in mice subjected to moderate hypoxia^91^ and in vitro studies implicated mechanisms involving intracellular Ca^2+^ regulation, several MAP kinase pathways and modulation of apoptosis regulators.^93^ On the other hand, isoflurane and several other anaesthetic agents have also been shown to interfere with neuroprotective mechanisms in response to hypoxia, inhibiting erythropoietin upregulation in the brains of mice.^94^ There is evidence that ketamine is also neuroprotective.^95,96^ Although there is potential for the anaesthetic to be a confounding factor, consistent application of anaesthetic protocols and randomisation will ensure that any neuroprotective effect will be controlled for. Even so, consideration of the magnitude of any systematic effect is still important as a very large effect could mask a more modest but real effect of a drug.

Anaesthetic agents can also have a direct effect on CBF, a key variable in the development of ischaemic lesions. The effect is dependent both on the agent selected and the dose administered. For example, of the inhalant anaesthetics, halothane causes a greater increase in CBF than isoflurane and sevoflurane.^97,98^ Injectable anaesthetics generally decrease CBF, with the exception of ketamine.^99^ Anaesthesia also results in changes in CBF indirectly because of effects on the respiratory system (see section 5 ‘Intraoperative care’). Choice of anaesthetic can therefore directly bias the results, but standardisation of methodology can reduce variability due to direct anaesthetic agent effects.

16. **The anaesthetic should be chosen on the basis of both welfare and scientific outcomes and should take account of species, strain and health status of the animals. Selection should involve the vet.**
17. **Sham-operated animals should receive exactly the same anaesthesia regimen for the same duration as the test group in order to control for effects of the anaesthetic on outcomes.**

### 3.3 Local anaesthesia

Local/regional anaesthesia is recommended to control pain induced by surgery. The use of a long lasting local anaesthetic at the surgical site can reduce pain and analgesic requirements. The anaesthetic should be injected prior to surgery, to provide a regional block into the area below the planned incision site. Once the hypodermic needle is in place, pull back on the plunger before injection to ensure that the needle has not entered a blood vessel by accident, as intravenous administration can be toxic.

Local anaesthetics act on Na^+^ channels to block nerve transmission. However, there are increasing reports of additional actions affecting other processes of relevance to stroke modelling, notably immunomodulatory effects.^100^ Inflammation and immune processes are involved in multiple aspects of stroke including underlying co-morbid disease and risk, injury-induced inflammation, and the major complication of stroke, systemic infection via stroke-induced immunosuppression.^101^ Local anaesthetics have potent anti-inflammatory effects (in some cases more so than nonsteroidal anti-inflammatory drugs (NSAIDs)) including inhibition of cytokine production and leukocyte trafficking, activation and phagocytic activity.^100^ They may also have anti-microbial properties.^102^ The effects are shared among the most commonly used agents, with the notable exception of ropivacaine (S-enantiomer specifically), which has relatively weak anti-inflammatory activity.^100,103,104^ Avoidance of inadvertent intravenous injection should minimise complications arising from the above effects. In addition, ropivacaine may be a good choice in general (see also desirable pharmacokinetic properties below) and particularly when analysis of immune influences on stroke is the specific objective.

Lidocaine-containing creams (e.g. EMLA or LMX4) can be used on the tail when i.v. catheters are inserted for venous access (e.g. anaesthesia, fluids) and also on the ear bars when rodents are placed into stereotaxic frames. LMX4 is a liposomal formulation that facilitates the extent of penetration into the skin in people, and has a shorter onset of action (www.lmx4.co.uk).

Types of local anaesthetic agent and their pros and cons are summarised in Table 2.

18. **Local anaesthesia should be used prior to incision during surgery, particularly if other types of analgesia are not being provided, and with knowledge of local anatomy to ensure that it is applied in the appropriate area.**

**Table 2.** Commonly used local anaesthetics.

	Dose (mouse)	Dose (rat)	Comments
**Injectable**			
Ropivacaine	Infiltrate into surgical area at up to 2 mg/kg		Ropivacaine less commonly used and weaker but long lasting and less likely to be toxic from inadvertent intravenous administration than bupivacaine or lidocaine. Bupivacaine is long lasting, lidocaine short acting. Ropivacaine has the weakest anti-inflammatory effects
Bupivacaine	Infiltrate into surgical area at up to 2 mg/kg		
Lidocaine (lignocaine)	Infiltrate into surgical area at up to 10 mg/kg		
**Topical**			
EMLA	Apply to clipped skin 20–30 min prior to anaesthetic effect being required		2.5% lidocaine/2.5% prilocaine emulsion
LMX4	Topical application to clipped skin as for EMLA		4% lidocaine in liposomal formulation. More rapid acting, longer lasting and less likely to enter the systemic circulation than EMLA.

The data in this Table are from the text of section 3.3 ‘Local anaesthesia’ or from Laboratory Animal Anaesthesia.^88^

### 3.4 Analgesia

Since all rodent stroke models involve some form of surgical intervention, post-operative pain can be anticipated. Pain may also occur as a result of the ischaemic lesion. Pain is frequent after stroke (for example, headache is reported by up to 38% of acute stroke victims)^105^ but it is not a predominant feature of human experience, and would be treated if it occurred. Uncontrolled pain does not make the animal model more similar to the human condition under study. EU Directive 63/2010 requires that analgesia is given unless it interferes with the scientific outputs. Ethically, analgesia should be administered to all animals in a study as pain is a predicted outcome. This prevents pain or pain relief becoming a confound in the experiment because the analgesia is provided systematically as part of the experimental protocol, i.e. both test and control groups receive it. Not controlling post-surgical pain in animal models induces stress and additional uncontrolled variables in the experiment; animals experiencing pain will not groom, eat, drink, or sleep normally.^106–109^ Some analgesic agents have been shown to have measurable effects on infarct size and growth of the lesion (e.g. buprenorphine^110^). However pain and the inflammatory response to tissue damage, and the more generalised surgical stress response, also interact with lesion induction. It may therefore be preferable to control pain and reduce the uncontrolled effects of tissue trauma, to attempt to reduce variability within and between treatment groups.

In the case of drug efficacy studies, other factors, such as competition of transport mechanisms, plasma protein binding or metabolic pathways may influence the selection of both analgesic and anaesthetic agents. It should be possible to identify agents with minimal potential interactions, and a decision to withhold analgesia should only be based on strong, reproducible evidence that the drug under study has a defined interaction with all available analgesic agents and that the assessment of efficacy would be compromised if analgesics were administered. Equally, in mechanistic studies, withholding analgesia should only be on the basis of solid evidence that the process under investigation cannot be studied adequately in the presence of the analgesic agent. In both cases, all potential means of pain relief should be considered.

As with anaesthetic agents, the effects of analgesic agents that might interact with study objectives are dose-dependent. It is therefore important that appropriate doses of agents are administered for an appropriate period (i.e. the doses needed to effectively control pain). This requires careful and accurate assessment of the presence of pain and its severity (see section 6.2 ‘Pain assessment in stroke models’). Pain assessment also needs to be repeated at intervals in order to determine the need for additional doses of analgesic agents.

When selecting an analgesic agent, the specific objectives of the experiment should be considered alongside the mode of action of the analgesic so as to minimise the impact of the broader effects of analgesic agents. For example, many analgesics, NSAIDs in particular, relieve pain primarily by inhibiting production of inflammatory triggers of nociceptors, e.g. via cyclooxygenase (COX) inhibition. Opioids, while being strong analgesic agents, also have immunomodulatory properties with considerable evidence showing immunosuppressive effects.^111–113^ Buprenorphine has a more favourable immune profile relative to other opioids (e.g. fentanyl, morphine) and thus may be a better choice.^113^ Of note, a recent study showed that buprenorphine reduced pain scores without affecting neuroimmune responses in a mouse model of meningitis.^114^ As noted above, inflammatory/immune processes are involved in multiple aspects of stroke aetiology and pathology (independently of pain pathways) and stroke-induced infection, which worsens outcome, is common in patients and observed in mice.^115^ Achieving pain relief without immunosuppression is therefore desirable, particularly in studies directly assessing immune function in stroke.

In terms of timing, analgesics should be administered before noxious stimulation occurs. This may prevent central sensitisation and also reduce the severity of the responses to tissue injury, reduce nociceptor activation and prevent peripheral sensitisation. It is recommended to integrate analgesic administration with the anaesthetic protocol. The animal’s level of pain and need for pain relief should also be assessed objectively (section 6.2 ‘Pain assessment in stroke models’) and additional analgesic should be used if required. It can be given to all animals at the same dose and timings (including sham-operated and animals not exhibiting pain behaviour) to avoid the effects of the analgesic agents becoming an additional uncontrolled variable in the study. Alternatively if different doses are used in different animals (to avoid pain being a confounder), it needs to be specifically integrated into the design and analysis of the experiment, and reported appropriately in the publication. Animals should be assessed for pain in between analgesic doses, to ensure that the regime is effective.

Analgesia should be given by the least stressful route of administration, and voluntary consumption of individual doses of analgesics in a palatable base has been shown to be effective (e.g. in flavoured gelatine).^116^ However, medication of food or water has more variable effects, and it is important to ensure that the analgesic is reliably consumed, in sufficient quantity.^117^ For example, intake of analgesics in water may be unreliable for animals that are less mobile, or where the taste of the water is altered by adding the drug. Administration in food and water may be ineffective, since food and water consumption are greatly influenced by diurnal rhythms, and animals may receive ineffective doses during the light phase of their photoperiod.^117^

Types of analgesics and their pros and cons are summarised in Table 3. As with anaesthetic agents, considerable variation between strains, ages and sexes in analgesic doses is known to occur,^118^ so selection of an effective dose regiment will require use of pain assessment.

19. **Pain is a variable which needs to be controlled. Pain relief must be used unless there are good scientific reasons not to, supported by solid, reproducible evidence.**
20. **The analgesic drug should be selected in consultation with the vet, based on the objective of the study, the specific stroke model and the type and timing of outcome measures.**
21. **The animal should be assessed for level of pain post-operatively, to ensure that the analgesic regime is effective and to minimise the risk of any unnecessary medication or side effects.**
22. **All animals should either receive the same doses of analgesics to avoid pain relief being a confounder, or the experimental design and analysis should account for animals receiving different doses. This should be reported explicitly in publications.**
23. **Analgesia should be given by the most reliable and least stressful route. If there is doubt about oral consumption, analgesics should be given parenterally.**

**Table 3.** Commonly used analgesics.

Mechanism	Analgesic	Dose (mouse)	Dose (rat)	Comments
NSAID	Carprofen	10 mg/kg s.c. (24 h duration)	2–5 mg/kg s.c. (24 h duration)	Carprofen affords 24 h relief and can be given by injection. COX-blockers interfere with inflammation processes. Can produce a protective effect per se depending on dose and duration
	Meloxicam	5–10 mg/kg p.o. or s.c.	0.5–1 mg/kg p.o. or s.c.	Meloxicam affords 24 h relief and can be given by injection or orally. COX-blockers interfere with inflammation processes
	Ibuprofen and aspirin	Not recommended		Poor control of dosing when given in drinking water, especially aspirin which is poorly soluble
Opioid	Buprenorphine	0.05–0.1 mg/kg s.c. 8 hourly. Given before animal is conscious because of slow onset (40 min)	0.05–0.1 mg/kg s.c. or p.o. 8–12 hourly. 0.5–1 mg/kg orally	Relative to other opioids least affects inflammatory processes, but inhibits glutamate release and affects key stroke outcome measures. Buprenorphine can be given by injection or orally
Other	Paracetamol	200 mg/kg once daily	50–150 mg/kg twice a day	Can be given orally as a palatable preparation. Poor control of dosing when given in drinking water if animals do not drink post-operatively as is often the case

The data in this Table are from the text of section 3.4 ‘Analgesia’ or from additional references.^88^

## 4 Aseptic surgical techniques

Aseptic technique is essential for both scientific and animal welfare reasons. Infection, as a result of poor aseptic technique and contamination may lead to inflammation, pain and delayed recovery, which may well compromise the experimental outcome and cause avoidable suffering.

While the goal of aseptic technique is to minimise the risk of infection through surgical contamination, it is also important to recognise that infection may be a natural part of the response to stroke that is not due to contaminating sources. Infection occurs in up to one-third of stroke patients with bacterial pneumonia the single most common cause.^119^ Recent studies have also shown spontaneous bacterial pneumonia occurring in rodent models of stroke.^120–122^ The mechanisms are incompletely understood but stroke-induced suppression of some aspects of systemic immunity may be important.^115^ Importantly, contamination from external sources is not the source of the infection in these cases but is most likely from increased invasiveness and/or displacement of commensal microbes (e.g. aspiration pneumonia). A recent study has also shown that bacterial lung infection after experimental stroke can result from increased stroke-induced gut permeability and bacterial translocation.^123^ This is consistent with human stroke studies where both respiratory and gastrointestinal dwelling commensal bacteria have been identified as causative agents in stroke-associated pneumonia.^124^ Thus, if signs of infection are observed, care should be taken in interpreting the cause/source. Nonetheless, given that many animals that have undergone stroke induction, notably those with large MCA territory infarcts, will be in at least a partially immunocompromised state, the need for aseptic technique is further underscored. Extra consideration of the handling and housing of potentially immunocompromised mice may also be necessary.

Prophylactic administration of antibiotics after stroke in human patients did not reduce the incidence of pneumonia or improve outcome compared to standard reactive treatment^125,126^ suggesting that routine prophylactic administration of antibiotics in animal stroke models may be similarly ineffective. Their use is usually unnecessary if surgery is performed aseptically, and their presence may interfere with the experiment (e.g. through alterations to microbiota composition. See also section 2.7 ‘Factors influencing host microbiota composition’). If antibiotics are used, as with analgesics, all animals should be treated identically so that this factor is systematic and controlled for.

### 4.1 Planning for surgery

Prior planning and attention to detail is essential for all surgical procedures and should encompass the preparation of instruments, consumables, facilities, the surgeon and the animal. Only the basic requirements are listed below insofar as they apply to rodent stroke models, and more detailed information is contained, for example, in the Laboratory Animal Science Association (LASA) Guiding Principles^127^ and in the video tutorials from the Procedures With Care website (www.procedureswithcare.org.uk).

It is important to ensure that the facilities are of an appropriate standard for stroke surgery before beginning any procedure on a live animal. The operating theatre and preparation areas should have all unnecessary equipment and other items removed and should be cleaned thoroughly between batches of animals. There must be appropriate training and supervision in place for new surgeons and a peri-operative care plan should be agreed with the veterinary and animal care staff before surgery commences.

Ensuring adequate standards of both asepsis and animal monitoring for a surgeon working on their own is very challenging. The surgeon is already concentrating on an involved manual procedure, making it difficult to monitor the animal's vital signs in a meaningful way. Achieving asepsis without a surgical assistant requires a high degree of organisation, and can result in more consumables being used and prolonged surgical time (Paul Flecknell, personal communication). This can increase the potential for adverse effects in the animal, and the variability in the experiment. Trained animal care staff can provide such assistance. Alternatively, in small research groups with limited resources, colleagues from other research groups can provide help during surgeries in exchange for similar help during their own operations. In addition to managing the anaesthesia and handling non-sterile equipment and consumables, assistants can also prepare the next animal for surgery and monitor animals that are recovering.

### 4.2 Preparation of surgical instruments and consumables

All surgical instruments and/or consumables should be checked well in advance of surgery with spares to hand in case of contamination or malfunction. This includes checking that supplies of anaesthetic gases are sufficient, particularly for long surgeries, and that any substances, solutions and medicines are not past their expiry dates. If diluted formulations are needed these should be prepared daily.

Instruments and surgical consumables such as swabs, needles and suture materials should be appropriately packed and sterilised by autoclaving before use. Autoclave tape can be used to indicate sterility. Ideally, a new set of sterile instruments should be used for each animal in order to avoid cross-contamination between animals. If several animals are undergoing surgery during a single session, then the choice is between having the right number of sterile surgery and consumable kits ready beforehand, or sterilising instruments after each use before starting to prepare a second animal. Sterile disposable instruments can be a cost-effective alternative if autoclaving is not available locally.

Bead sterilisers can be used during surgery to sterilise the tips of instruments, or between animals during a session of surgeries, but should not be regarded as an alternative to autoclaving. In case of contamination, the instruments/consumables should be replaced between surgeries and sterile instruments should be prepared for each session of surgeries. Other methods of sterilisation, such as ethylene oxide or irradiation, can be used for equipment which cannot be exposed to steam, but alcohol or disinfectant are not recommended, as these do not provide adequate sterilisation.

### 4.3 Preparation for the surgeon and assistants

‘Non-scrubbed’ surgical assistants must not touch sterile instruments, drapes or consumables. The surgeon and assistants should wear a head cover and a mask. The mask should be applied before scrubbing, gowning and gloving to cover all facial hair. Having taken off any watches or jewellery, the surgeon should perform a thorough scrub of hands and nails using a commercial product designed for the purpose, such as Hibiscrub, following the manufacturer’s instructions. With the help of an assistant, the surgeon should put on a sterile, long-sleeved operating gown. Sterile gloves should be opened preserving sterility and worn over the cuffs of the gown.

Throughout the procedure, the surgeon must be careful to avoid touching non-sterile items such as the table, the animal, experimental or anaesthetic equipment, or the operating lights. If contamination occurs, gloves should be replaced. Risk can be minimised by wiping surfaces with disinfectant wipes prior to surgery and covering with sterile drapes. Throughout surgery, an assistant should be available to open the outer packing of sterile items such as sutures or scalpel blades, move the animal, and adjust the table and any non-sterile equipment and to assist with monitoring the depth of anaesthesia. If the surgeon needs to touch a non-sterile surface for example to make fine adjustments to an operating microscope, the adjustable knobs can be handled though a large sterile swab, which is then discarded. Alternatively, it can be covered with suitable sterile material, such as sterile foil or plastic covering, before surgery starts.

### 4.4 Preparing the animal for surgery

Animals should be subject to a general observation (e.g. general behaviour and condition of their skin and fur) to ensure that no sign of illness or wounding by cage mates is present before surgery.

The animal should be anaesthetised in a procedure room which is separate from holding rooms which house conscious animals. Following the induction of anaesthesia, sufficient hair must be clipped from the surgical site, using an electric clipper. This should be done at a distance from the operating table or in an adjoining room, to avoid contaminating the operating area with hair, dander and associated sources of microbial contamination. The shaved area should be large enough to allow adequate skin preparation, prevent hair ingress into the incision during surgery and afterwards during wound healing. The size of the clipped margin around the incision site should be the minimum compatible with achieving the objectives outlined above.

The skin should be cleansed and then prepared with a suitable topical solution (e.g. chlorhexidine, diluted in alcohol or water, or povidone-iodine); alcohol alone is not suitable as a skin disinfectant. These solutions should not soak the whole animal, but should be applied to the clipped skin in accordance with manufacturer’s instructions to reduce microbial contamination to minimal levels in the prepared area.

### 4.5 During surgery

The use of transparent disposable drapes over the animal can aid anaesthetic monitoring. Sterile drapes should be big enough to cover unprepared parts of the animal and adjacent surfaces. Instruments should be placed on a sterile drape or tray within the sterile field; the drapes should cover a sufficient area for the surgeon to lay out and use instruments and suture materials without accidentally contacting non-sterile items or surfaces.

### 4.6 Monitoring outcomes of surgery

Standards should be monitored to assess the surgeon’s performance. Acceptable success rates for each type of surgery should be agreed on within the team, and surgeons who do not achieve it should either receive additional training or refrain from operating on animals. To that end, each surgeon should keep accurate records of deaths during general anaesthesia and surgery, wound breakdown, and requirement for intervention.

24. **Aseptic surgical technique is essential.**
25. **Antibiotics should not be used prophylactically unless there is a justified case.**
26. **The surgeon should work with an assistant.**
27. **Surgeons’ performance should be monitored and reviewed.**

## 5 Intraoperative care

Attention to care of the animal during surgery is as important as the technical procedure itself in ensuring good quality of outcome for both the animal and the science. At the most basic level, support requirements for anaesthetised rats and mice are warmth and oxygen.

### 5.1 Preparation for anaesthesia

The blink response will be lost under general anaesthesia so it is important to protect the animals’ eyes. Protective gel/drops (e.g. Viscotears, Lipolac, Lacri-lube ointment or similar) will prevent drying of the cornea during anaesthesia, and should be applied immediately after clipping. If anaesthesia is prolonged, re-application may be required after 30–40 min.

### 5.2 During the surgery

#### 5.2.1 Body and brain temperature

Temperature is particularly important in stroke models, as hyperthermia (elevated body temperature due to failed thermoregulation) has been shown to be a determinant of poor outcome following experimental stroke.^128^ In fact, brain cooling, known as therapeutic hypothermia, has been shown to be an effective neuroprotective strategy following ischaemic stroke.^129^

All anaesthetics result in hypothermia, because of effects on thermoregulation, by depression of metabolic rate, and by peripheral vasodilation. Most experimental stroke studies are performed in rodents and due to their relatively small size and relatively higher surface area to body weight ratio, they rapidly lose core temperature unless measures are taken to prevent this. Small rodents show a 1–2℃ fall in core temperature immediately following induction of general anaesthesia and core temperature will continue to fall. Maintenance of core body temperature should be viewed as a central part of anaesthesia management to prevent hypothermia. This should ideally be done through a feedback-controlled system, which monitors the core body temperature of the animal and adjusts the temperature of the heating device to maintain the desired body temperature. Heating mats and pads may be less effective when animals are placed in a stereotaxic frame, since the area of the animal in contact with the heat source can be greatly reduced. Use of insulating material over the thorax and abdomen, and tucking the tail under the animal’s trunk can help reduce heat loss. Careful monitoring of body temperature is essential to ensure these measures are effective. It is common practice to monitor the efficacy of warming devices by recording rectal temperature; however this does not always accurately reflect brain temperature in man or animals.^130,131^

Special consideration is required for obese animals as heat from heating pads does not dissipate as well because fat is less well perfused. Problems can be avoided by increasing the thickness of material between the heating pad and the animal and by additional monitoring of skin temperature (with an upper limit of 41℃).

28. **During general anaesthesia and in the immediate post-operative period, the animal’s body temperature should be maintained by insulation or supported by a heating device, with a feedback heating system that cuts out when normal body temperature is reached.**
29. **Additional care and monitoring of body temperature may be needed for obese animals.**

#### 5.2.2 Cardiorespiratory effects of *anaesthesia*

All anaesthetic regimens have depressant effects on the respiratory system, resulting in an increase in arterial carbon dioxide tensions (PaCO_2_). CBF shows a linear relationship with PaCO_2_, increasing as PaCO_2_ increases, so long as blood pressure remains within the normal physiological range.^132^ These effects can be controlled by endotracheal intubation and ventilation of the animal. Intubation and ventilation also enable easy manipulation of the inspired oxygen concentration (FiO_2_), another factor than can influence the ischaemic lesion.

In order to maintain tissue oxygenation, and crucially, to avoid variation in the induction and progression of the cerebral infarct, oxygen can be delivered to the animal via a nose cone, face mask or by oral intubation, whether anaesthesia is by gaseous or injectable agent. When induction of ischaemia can be achieved rapidly (e.g. in less than 30 min), and when the anaesthetic protocol does not result in significant hypercapnia, the additional time needed for intubation may not be warranted and a nose cone or face mask may represent the most straightforward approach. For prolonged anaesthetic protocols (over 30 min), or protocols when it is advisable to control arterial carbon dioxide and/or oxygen tensions, then oral intubation with artificial respiration should be considered as this will enable physiological stability of the animal to be maintained. In rats that are intubated and artificially ventilated, MCAO surgery results in smaller infarct volumes and reduced mortality compared to spontaneously breathing animals.^133^

Endotracheal intubation is feasible in rats and a wide range of techniques have been described.^88^ Although more challenging, it is also possible in mice. Intubation can be carried out using an ‘over-the-needle’ catheter as an endotracheal tube and simple improvised or specialist equipment.^134,135^ Following intubation, the animal can be ventilated to maintain normocapnia (normal arterial carbon dioxide pressure) and prevent hypoxia. Species-specific ventilators should be used and ventilators suitable for rats and mice are available from a number of manufacturers. During prolonged anaesthetic protocols with artificial ventilation, problems with increased secretions from lungs or mucus plugs blocking intubation tubes can be avoided by prophylactic administration of atropine (0.05 mg/kg subcutaneous (s.c.) or intraperitonal (i.p.)) or glycopyrrolate (0.5 mg/kg intramuscular (i.m.)).^88^

Blood oxygenation can be monitored invasively by blood gas analysis of arterial blood samples (partial pressure of oxygen, PaO_2_ in mmHg) or non-invasively by pulse oximetry (oxygen saturation of haemoglobin, SaO_2_ in %). Normal levels of blood oxygenation for rats and mice breathing air are ∼82–94 mmHg^88^ and 95–97% SaO_2_. The normal range for carbon dioxide in the blood is 35–45 mmHg^136^ and this can be assessed both by direct measures from arterial blood or by use of capnography. Although blood gas analysis can be undertaken using relatively small volumes of blood (50–100 µl), this is too great a volume to allow repeated sampling in rats, or even single samples in mice. Capnography, even using specialised apparatus, does not give as accurate a measure of arterial carbon dioxide in rodents in comparison to larger species, but it can enable consistent levels to be maintained within and between studies.^88^

Stroke models are also affected by brain oxygen tension, both at the time of induction of the stroke, and subsequently. Brain tissue oxygenation will be affected by a number of factors, including the use of oxygen as the carrier gas for volatile anaesthetics, or as a means of preventing hypoxia when injectable anaesthetics are used. If medical air is used as a carrier gas, spontaneously breathing animals will become hypoxic, but normoxia (and normocapnia) can be maintained by use of intermittent positive pressure ventilation. Manipulating the inspired oxygen content in spontaneously breathing animals can enable more physiologically normal oxygen content to be maintained, but these animals will become hypercapnic. An FiO_2_ of 100% oxygen will give PaO_2_ values in excess of 300 mmHg when the animal is artificially ventilated, and 200–300 mmHg in most spontaneously breathing animals. An O_2_/N_2_O (e.g. 30:70) mix or 28–30% O_2_ will give PaO_2_ values around 100 mmHg with artificial ventilation but in spontaneously breathing animals, hypoxia may occur. If endotracheal intubation is not undertaken, then oxygen can be delivered to the animal via a face mask or by nasal intubation, whether anaesthesia is by gaseous or injectable agent. A face mask is the most straightforward approach for brief surgical protocols.

The effects of anaesthetics on both respiration and circulatory function are generally dose-dependent, so minimising the dose of anaesthetic by use of adjuncts such as local anaesthetics to provide additional analgesia may be of value. Use of nitrous oxide to provide additional anaesthetic and analgesic effects is of limited benefit in small rodents, since minimum alveolar concentration (MAC) value (a measure of anaesthetic potency) is high (indicating low potency) in both rats and mice.^137,138^ However, 70% nitrous oxide can produce a 30% reduction in sevoflurane MAC in mice,^138^ so this agent may have some value as an anaesthetic adjunct. Use of N_2_O mixtures with oxygen, to reduce the FiO_2_ can be used, but N_2_O also has a wide range of other effects,^139^ so nitrogen or air mixtures with oxygen are preferable.

#### 5.2.3 Physiological monitoring during anaesthesia – General considerations

Decisions as to the nature and extent of monitoring animals during anaesthesia depend on the level of interventions, equipment access and duration of surgery and the scientific objectives of the study. The potential benefits of invasive monitoring need to be balanced against the potential for causing additional post-operative pain or discomfort (e.g. following arterial cannulation). Monitoring basic physiological parameters is essential for ensuring a controlled, stable anaesthesia that is safe and effective for the animal and is likely to improve reproducibility of the study. Basic requirements should include regular checking for respiratory movement, normal pink colouration of the extremities and adequate depth of anaesthesia (e.g. assessed using the pedal withdrawal reflex), which allows early identification of problems than can be quickly corrected. It is also important to keep a record of these and surgical activities for each experiment (e.g. start/end of procedure, significant interventions including drug administrations, any problems encountered).

Clinical monitoring can be challenging but specialised monitoring equipment for small rodents is relatively inexpensive, can record a rodent’s rapid heart rate and be linked to a computer to record study data. Non-invasive methods of monitoring such as pulse oximetry should be used to ensure physiological stability under anaesthesia. This technique measures cardiorespiratory function via the tissue oxygen saturation and heart rate with a sensor placed on the paw, tail or tongue. As mentioned earlier, other non-invasive methods of monitoring, such as capnography and systolic blood pressure (via tail cuff plethysmography) are technically more difficult, due to the small size of the animal. As mentioned above, it is often possible to use capnography to standardise respiratory function within and between studies, and this is strongly recommended when inducing stroke, and subsequently in rodent fMRI studies where stability of PaCO_2_ is also important.

Mean arterial blood pressure, heart rate, blood gases and pH can be monitored invasively with arterial (e.g. femoral) cannulation. These enable accurate measurement of the animal’s status but repeated blood sampling for blood gas analysis will be limited by the small size of rodents.

Individual animals may vary in precise values for clinical parameters such as heart rate, so trends in observations are more helpful in identifying significant deviations from normal. If the animal is anaesthetised and intubated, variables such as PaO_2_, PaCO_2_ and O_2_ can be controlled to be within the physiological range by altering ventilator settings (tidal volume and respiration rate) and percent oxygen delivery. Small adjustments to the depth of anaesthesia can help maintain a stable blood pressure. Control of these variables therefore contributes to improved reproducibility of stroke outcome measures.

30. **Monitoring of respiratory and cardiovascular parameters is essential for animal safety and the reproducibility of study methods.**
31. **Minimum parameters to monitor are depth of anaesthesia, respiratory rate and temperature. Surgical activities should also be recorded.**
32. **Pulse oximetry is recommended as SaO_2_ is a good indicator of tissue oxygenation and heart rate is difficult to monitor otherwise.**
33. **Invasive monitoring is useful for experiments carried out under terminal general anaesthesia or for specific cases justified by the study needs.**
34. **Intubation and artificial respiration should be considered for experimental protocols involving induction of ischaemic lesions, particularly for those lasting longer than 30 min.**

#### 5.2.4 Fluid balance

During anaesthesia animals will lose body fluids, via a variety of routes. Some agents (e.g. medetomidine and xylazine) cause a diuresis and fluid loss can be significantly increased. During the post-operative period, fluid intake may be reduced, especially in animals showing clinical effects of stroke.

Fluids should be administered to compensate for these intraoperative and post-operative fluid losses. Fluids should be warmed to body temperature, and be sterile and isotonic. Saline or Hartmann’s solution is suitable and given at 1 ml/100 g bodyweight (i.v., s.c. or i.p.) repeated every 1–2 h of anaesthesia.

35. **Animals should be administered fluids pre-emptively to prevent dehydration during surgery.**

## 6 Post-operative care

Following induction of cerebral ischaemia a number of clinical signs will be present. Animals should be monitored closely and assessment of clinical signs should take into account the severity of the ischaemia induced along with the health status of the animals pre-ischaemia as, for example, premorbid animals (e.g. hypertensive, obese) can show accentuated adverse effects compared to normal young, healthy animals. Animals should be monitored frequently (see below) to identify any adverse effects which would require further intervention. Non-invasive home cage monitoring systems can be used to record locomotor activity.^140^ Clinical assessment sheets should be used to allow monitoring of expected and unexpected adverse effects.

To prevent hypothermia, temperature in the post-operative room should be monitored. It should be warm and local sources of heat such as heated cabinets or heating pads should be used.

### 6.1 Monitoring of animals

Animals should be assessed at a time point considered reasonable for recovery from the anaesthesia. This will vary depending on the anaesthetic agent used, along with the species and health status of the animals. It is recommended that post-stroke animals are monitored at least four times a day at regular well-spaced intervals, during the first 48 h. Beyond 48 h, animals should be monitored at least once a day but the frequency of monitoring should be increased if co-morbid animals are used and if animals are showing any clinical signs requiring intervention. Importantly, clinical assessment sheets should be used to record such monitoring and be kept with the animals. Monitoring should be done with minimal handling but sufficient to allow adequate welfare determination in order to identify those animals which (i) have reached a humane endpoint and should be euthanised, (ii) are at highest risk of not surviving and (iii) may require extra monitoring and/or intervention such as extra fluid supplementation. Clinical signs that may present following induction of cerebral ischaemia have been characterized as green, amber or red, as detailed below (Table 4). The guidelines apply to adult rodents from standard, commonly used strains and may need adjustment under other circumstances (e.g. for aged, young or obese animals).

**Table 4.**
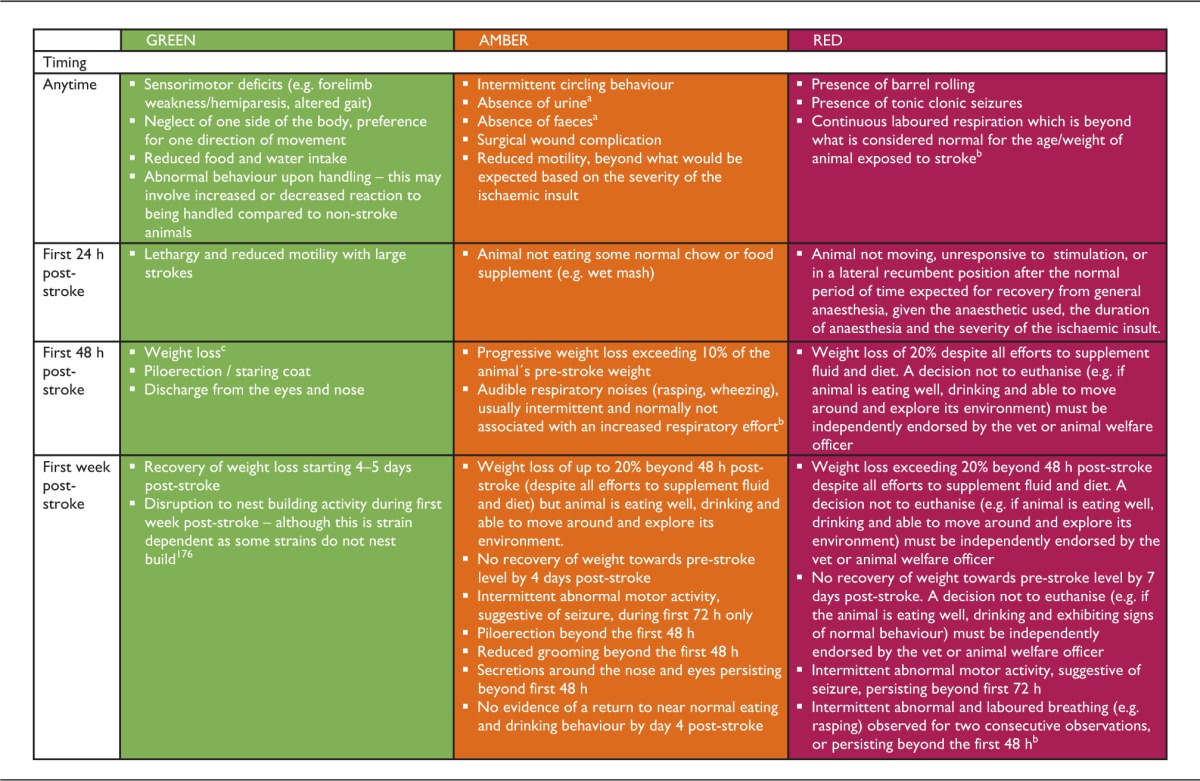
Signs to monitor after experimental stroke surgery in rodent models.

a These would typically be observed in singly housed animals to assess if the animal is eating and drinking sufficiently. However, group-housing has been shown to aid recovery (see section 2.4 ‘Social housing post-stroke’). In group-housed animals, observing eating/drinking habits, monitoring body weight and whether the animals defecate/urinate during handling can be used to assess whether each animal is eating and drinking sufficiently.

b Abnormal breathing which is beyond what is considered normal for the age/weight and characteristics of animal exposed to stroke. For example, obese animals may exhibit noisier rasping respiration than non-obese animals.^180^ Intermittent wheezing (amber sign) can be seen in the first 24 h after MCAO and is normally associated with intubation and/or long or repeated anaesthesia, most likely due to accumulation of respiratory secretions and/or minor laryngeal trauma. It should resolve within 24–36 h. Respiratory distress (red sign) may be a result of pulmonary oedema associated with the MCAO^181,182^ or severe laryngeal trauma at the time of intubation.

c Weight loss after stroke is common, both in humans and in rodent models, and can be explained principally by dehydration, impaired feeding, inactivity and paralysis. However, other factors such as neuroendocrine sympathetic activation, fever and inflammation also contribute to metabolic imbalance and an increased catabolic drive leads to tissue wasting of both fat and muscle, depleting energy stores and leading to functional decline. Rodent models typically demonstrate a dramatic weight loss after stroke surgery, which normally starts recovering after 4–5 days. The amount of weight lost during that period is tightly correlated with the size of the infarct^179^ (see Figure 1).

GREEN – clinical signs similar to those seen in clinical stroke are expected to be present after a focal ischaemic insult as it results in brain injury and swelling. For some studies where scientific endpoints can be achieved by small infarcts, signs may be reduced in severity or absent. It is anticipated that the majority of animals will not display all of the clinical signs, and any clinical signs should start to show improvement by 48 h post-stroke.

AMBER – the presence of any of the signs in the amber column below requires an increase in the frequency of monitoring and appropriate intervention, such as consulting the vet or implementing some of the recommendations contained in this paper. If any of the clinical signs exceed the limits stated, the animal should automatically move to RED status.

RED – the presence of any of the signs in the red column below requires immediate euthanasia of the animal via an approved method of humane killing (in the UK, Schedule 1 or other licensed method).

36. **The experimental study plan should include details of planned post-operative intervention and assessment points. This should be devised in consultation with veterinary and animal care staff.**
37. **Animals should be monitored frequently (at least 4 times a day at regular interval during the first 48 h post-stroke) using a traffic light system (see Table 4) and should be humanely killed if they reach a pre-defined humane endpoint (red status).**
38. **Monitoring frequency must be increased if co-morbid animals are used and if animals are showing any clinical signs requiring intervention (amber status).**
39. **Monitoring duration should be long enough to ensure eating and drinking behaviours are observed.**
40. **Clinical assessment sheets should be completed each time animals are monitored – such sheets should remain with animal cages to ensure record of observations and consistency of care.**

### 6.2 Pain assessment in stroke models

We currently have very limited validated methods of pain assessment in rodents. Clinical assessment of animals are usually non-specific for pain, however they may be of value in allowing a structured evaluation of the overall state of the animal. The introduction of facial expression as a reasonably rapid and reproducible means of assessing pain in rodents offers advantages in ease of use, requiring minimal training of the observer. These grimace scales are based on changes in a number of ‘facial action units’, such as narrowing of the eyes (orbital tightening) or changes in the position and shape of the ears and whiskers. They have been developed for both rats^141,142^ and mice^143^ and validated for post-operative pain, e.g. in experimental myocardial infarction and other surgical procedures.^144–146^ However, neither the grimace scales nor other pain behaviours have been investigated and validated in stroke models, and further research is necessary to determine their sensitivity and specificity in these models. The methods used to assess pain in stroke models should be described explicitly in publications, to allow a clearer picture to emerge.

### 6.3 Supplementary fluids and diet

To optimise welfare, physiological stability and help minimise pain and discomfort, such as from needing to reach for food and water, animals should be kept adequately hydrated. Various methods are available (e.g. subcutaneous fluid injections, gel rehydration packs, soft diet). Dietary supplementations, if not part of the normal diet of the animal pre-operatively, should be introduced before surgery in order for the animals to overcome any neophobia.

Subcutaneous fluids should be administered prophylactically before stroke surgery to avoid dehydration. Post-surgery, additional methods of rehydration should be used routinely for severe stroke, but for less severe models, an assessment of the need should be made before giving additional fluids. Animals should be weighed frequently because the majority of weight that is lost after surgery is usually due to loss of water: this must be replaced. Signs such as sunken eyes or skin tenting following a skin pinch indicate that dehydration is already substantial; rehydration should be provided well before animals display such signs.

In order to maintain hydration by subcutaneous injection, local guidelines regarding volume limits should be adhered to and advice sought from the relevant person (e.g. animal welfare or veterinary staff). An example of a standard approach would be maintenance fluids of 2–4 ml/kg/h (or 80 ml/kg/day) using sterile and isotonic fluid (e.g. saline) which should be warmed to room temperature prior to use in order to avoid irritation if fluids are too cold. The route for rehydrating an animal depends on the circumstances. In an awake animal, the most effective route of hydration is oral and fluid intake can also be maintained by providing wet mash and/or recovery gel. The subcutaneous route can be used if the animal is not swallowing well. During anaesthesia, fluids can be administered intravenously or, if there is no venous access, intraperitoneally. For conscious animals, the intraperitoneal route is painful and requires firm restraint, thus a less stressful alternative is preferable. It is important to monitor the effectiveness of fluid intake/administration by, for example, observing animals for evidence of urination.

To encourage eating and provide fluid, chow pellets softened with water (‘mash’) can be provided in a plastic weighing tray and additional dry pellets can be scattered on the cage floor as animals may have difficulty retrieving the pellets from the hopper and/or drinking from water bottles after stroke. The soft food should be replaced at least once daily for seven days. If animals are group-housed after stroke as recommended, a sufficient number of soft chow trays and pellets should be put in the cage to avoid any competition for food. Providing wet food in the late afternoon fits with natural feeding patterns of nocturnal animals, and may be more effective, as the food is fresh at this time.

If body weight drops by 10%, then food supplementation such as Complan, or baby food can be tried. Also hand feeding animals should be considered to encourage weight gain after stroke surgery. The sugar content of any planned supplement should be considered as high sugar foods might increase blood glucose levels and exacerbate acute ischaemic damage.

**Figure 1.**
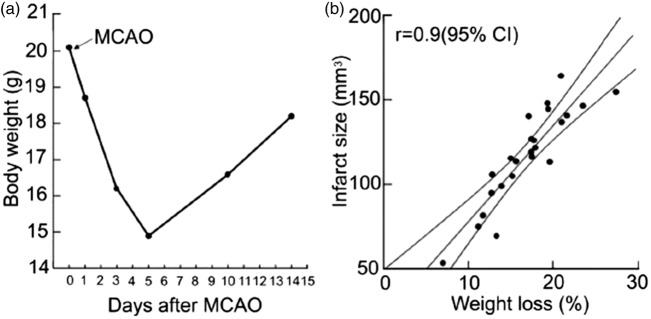
(a) Mean body weight after 60 min of middle cerebral artery occlusion (MCAO) in the BL6 mouse strain (*n* = 10). Note the dramatic drop and slow recovery of body weight, whereas nonmanipulated control mice gain 1 to 2 g per week. (b) Correlation of infarct size with loss in body weight 72 h after 60 min of MCAO. Note the very tight correlation between infarct size and loss in body weight. 95% CIs for the population mean (regression). Reproduced with permission from Dirnagl.^183^

Post-operatively, swallowing may be impaired by stroke so liquefied foods that might be inhaled should be avoided.

41. **Dehydration should be assessed frequently and treated post-operatively until the animal is seen to be drinking normally.**
42. **Additional hydration should be provided at least for the first two days post-surgery.**
43. **Animals should be provided with an appropriate post-surgical diet (e.g. wet mash). Softened food and loose pellets on the cage floor should be supplied for at least seven days post-stroke.**

## 7 Refinement for specific models

### 7.1 Intraluminal filament model

#### 7.1.1 Entry point of intraluminal filament when using the intraluminal filament model

In rats the filament can be introduced through an opening in the CCA, internal carotid (ICA) or the external carotid artery (ECA). The original approach via the ECA results in the ECA being cauterised and therefore disrupts the blood supply to the ECA territory, including facial musculature. This leads to significant additional weight loss, drinking impairments and confounds in behavioural measures. Avoiding permanent ligation or cauterisation of the ECA is a refinement; it prevents damage to the muscles of mastication and reduces weight loss, drinking impairments and reduces confounding side effects of surgery on behavioural outcomes.^148–150^ This can be achieved by introducing the filament via the CCA with either permanent ligation of the artery or careful sealing of the incision on filament removal^148,149^ which results in comparable outcomes.^148^ However, as variable stenosis of the CCA was reported when sealing an incision in the CCA,^149^ tieing off the CCA is recommended to reduce variation. The filament may also be introduced via the ICA.^150^ The use of these approaches does not alter the size or variability in infarct volume compared to entering via the ECA in rats and therefore are preferable particularly if behaviour is an outcome measure.

Filament insertion into the ECA or CCA is possible in mice.^151^ However, care should be taken as vascular anatomy is variable between mouse strains, with incomplete circle of Willis reported in some strains.^152–154^

However, if the CCA or ICA is tied off, the ipsilateral CBF may recover to approximately 80% of baseline.^151^ This approach may thus not be appropriate for a study looking at blood flow. However, this is not pathological – increased infarct volumes, or increased gliosis in comparison to the ECA model have not been reported. No pathology has been reported either in sham animals which have one carotid artery tied off.^148^

#### 7.1.2 Right versus left MCA occlusion

The side of occlusion can be altered and is often a matter of the surgeon’s preference. Left-handed surgeons tend to find that occluding the right MCA is easier and vice versa. However, it should be noted that the side of occlusion can affect experimental outcomes.^155^ In addition, in some cases, experimenters assess paw dominance before surgery and lesion the dominant hemisphere in each animal to give more robust behavioural deficits.

#### 7.1.3 Coagulating the occipital artery, superficial temporal artery branches and pterygopalatine artery

This helps to control blood loss and prevent collateral perfusion or retrograde blood flow. However, it alters blood flow to structures external to the MCA territory, which could impact on recovery of the animal and functional outcome measures.

#### 7.1.4 Waking the animal during the occlusion period

Waking the animal up during the occlusion period reduces the total exposure to anaesthetic agents. Some groups anecdotally report better recovery when doing this. Behavioural signs, such as circling, twisting when held briefly by the tail, and lack of response to whisker stimulation on the contralateral side can be used during this period for rapid assessment to confirm stroke. On the other hand, by keeping the animal on the operating table anesthetized for the duration of surgery, temperature can be more tightly regulated and other physiological parameters including CBF can be directly assessed during the ischaemic insult.

#### 7.1.5 Refinement to reduce mortality in severe strokes

A refinement for intraluminal filament-induced transient MCA occlusion in spontaneously hypertensive stroke-prone rats (SHRSP) has been reported, which reduces mortality and post-stroke associated weight loss.^156^ SHRSP rats exhibit many of the co-morbidities associated with stroke clinically including hypertension, altered glucose handling and elevated inflammatory signalling. The refinement involves an additional general anaesthetic, a cranial burr hole and durotomy six days prior to transient MCA occlusion and was identified by chance in an earlier study which required a general anaesthetic and stereotaxic injection of a substance one week prior to stroke.

Although recommended for intraluminal filament transient MCA occlusion in SHRSP, this refinement would be worth applying in any of the closed skull models where mortality is an issue.

#### 7.1.6 Choice of filament

Several suture materials and methods for blunting or coating filament tips have been used in filament MCA occlusion studies.^157–159^ The most appropriate will depend on a number of factors including animal species, strain, weight, surgical approach and vascular architecture. Key considerations include the flex and diameter of the main filament and the diameter and length of the tip coating.^160^ Flex and diameter of the main filament are important to enable passage along the kinks in the internal carotid as it passes through the skull. More flex is also likely to minimise the risk of puncturing the vessel at the base of the brain and causing a bleed as the force of pushing is not transmitted as strongly. Tip diameter and length will influence the occlusion success but may also affect the distribution of damage, instance of skull base subarachnoid haemorrhage and associated morbidity.^159–162^ While a thicker and longer coating could ensure more consistent occlusion, it may also occlude other deep vessels branching directly from the circle of Willis to subcortical structures, although this will depend on specific species and strain cerebrovascular architecture. The intraluminal filament occlusion model results in an occlusion duration-dependent increase in severity of cerebral hypoperfusion within, and surrounding the MCA territory.^154^ Beyond the MCA territory, regions such as the thalamus, hypothalamus and hippocampus may be affected as a consequence of the filament occluding more proximal arteries that arise from the ICA (e.g. anterior choroidal and hypothalamic artery^163^) or because animals have a hereditary incomplete circle of Willis. It is thought that damage to such areas as the hypothalamus compromises the stress and inflammatory responses and generates a hyperthermic response persisting following recovery.^164^ In general, the smallest diameter and shortest coating that produces consistent MCA territory injury should be used.

Until recently, most labs made their own filaments and considerable differences across labs were evident. However pre-made filaments in various sizes to suit different applications are now available from commercial sources (e.g. Doccol) and increasingly employed. Consistency afforded by commercial manufacture may contribute to improvements in reproducibility.

### 7.2 Embolic stroke

The intraluminal approach is also used to induce embolic stroke, the filament being replaced by a fine cannula, loaded with a pre-formed blood clot. The point of entry for the cannula is either the CCA or ECA as for the filament model and the tip advanced towards the origin of the MCA to release the clot. Although this is the most clinically relevant model, it is also one of the most difficult to achieve reproducibility in outcome measures even between surgeons in a single laboratory, and induces significant mortality (e.g. >30%).^165^ Stroke severity can be controlled by altering the composition and length of clot injected and the rate at which the clot is injected. Therefore, it would not be the model of choice to use for early studies of new stroke therapeutics, unless they were thrombolytics. However, if a therapy demonstrates efficacy with the non-embolic stroke models, it would be important to test it in combination with rtPA in the embolic model to confirm no adverse interactions with the current licenced therapy and to investigate any synergistic or additive effects.

### 7.3 Photothrombosis

Photothrombosis provides a more controllable model of blood clot-induced ischaemia mediated by generating a thrombus within cortical pial vessels. Cortical ischaemia has been induced by photothrombosis in various species including rats and mice. The procedure involves surgical anaesthesia, incision of the skin at the midline overlying the skull and then placement of a light source perpendicular to the location of infarct that is desired. In rats, skull thinning using a drill is done by some laboratories and not by others. A photosensitive dye, Rose Bengal, is injected by one of various routes: the intraperitoneal and intravenous routes are the most commonly used. Immediately thereafter, or after a delay, a light beam is shone through the thinned skull for a specified period. This initiates formation of a thrombus and local ischaemia (see Alaverdashvili et al.^166^). Various light sources may be used. Some groups use lasers which emit at a suitable wavelength for Rose Bengal. Others shine light from a wide-spectrum cold-light source via a fibre optic light source: the Schott KL 1500 LCD is very commonly used.

Care should be taken not to heat the skull and brain even when using ‘cold-light’ sources. Many cold-light devices use 150 W halogen lamps that are hot and can burn (due to emission of infrared and ultraviolet wavelengths). This is particularly true when illumination is used at the highest settings (e.g. when using an intensity of 3300 K colour temperature and setting ‘6E’ on the Schott KL 1500 LCD). Many groups now use illumination of 3000 K in rats to reduce mortality (Nicolas Lindau and Lukas Bachmann, personal communication) whilst another uses 3200 K using the Schott KL 200 light source (Antje Schmidt, personal communication). Finally, for rats, another group uses 3200 K plus a lower setting (5C, Schott KL 1500 LCD) plus a green insert filter to exclude infrared and ultraviolet wavelengths but to pass wavelengths of light that stimulate Rose Bengal (peak ∼560 nm) (Geralda van Tilborg, personal communication).^167^ For mice, some place the tip of the fibre optic directly on the skull^168^ and others lift it 2 mm above the skull (Geralda van Tilborg, personal communication). When using a cold-light source, we recommend that the target of the fibre optic is tested for temperature. Initially after turning on, the target may be cold but with time, intense illumination can cause burning.

Mannitol may be used to attempt to reduce swelling after photothrombosis in adult rats^169^ but anecdotally this is not effective in mice (Nicolas Lindau, Lukas Bachmann, personal communication). Mice with low body weights (and little body fat) (e.g. <20 g) appear to survive cortical ischemia by photothrombosis less well than mice with higher body weights (and more body fat). It may be useful to provide supplementary warmth during surgery, and post-operatively overnight (e.g. 25℃) and for several days thereafter (Michelle Porritt, personal communication).

### 7.4 Thrombin injection

A localised thromboembolic stroke can also be induced by the in situ injection of thrombin within the lumen of a distal branch of the MCA.^170^ The resulting thrombus produces a very reproducible cortical infarct in mice, although rapid spontaneous thrombolysis can be a source of variability. The blood clot can be successfully lysed by rtPA making this model suitable for the study of new thrombolytic drugs or drugs used in combination with thrombolytics. However, although the location and extent of the infarct is very reproducible, the infarct per se, is small and little or no neurological deficit can be detected. Therefore this model would be less useful to study functional outcome.

### 7.5 Endothelin-1

Endothelin-1 is a potent and long lasting vasoconstrictor peptide and therefore an ideal agent for inducing small, focal ischaemic lesions. Using a stereotaxic frame and co-ordinates, focal ischaemic lesions are induced by stereotaxic microinjection of endothelin-1 into specific neuroanatomical sites (e.g. white matter tracts such as the internal capsule) in adult or elderly rats.^171,172^ In rare cases, animals can exhibit ‘barrel rolling’ or seizures after injection of endothelin-1. This may happen if endothelin-1 accidentally enters the ventricles. In our experience the prognosis is not good and we recommend prompt, humane killing of any animals that display barrel rolling behaviour (see section 6.1 ‘Monitoring of animals’). Accordingly we recommend that endothelin-1 is injected into the brain via a route that does not impinge on the ventricles; oblique angles may be used to target deeper structures such as the internal capsule.^173^

### 7.6 Permanent MCAO by electrocoagulation in rat or mouse

Permanent occlusion of the main trunk of the MCA or its distal branches can be achieved by electrocoagulation. In this experimental model, the MCA is exposed by a craniotomy and opening of the dura mater. Using fine diathermy forceps, a current is passed through the artery which induces blood clotting, destruction of the artery walls and ischaemia distal to the electrocoagulation site. Sectioning the occluded part of the artery with microscissors, once electrocoagulation is complete, provides confirmation of complete occlusion. The severity and location of the ischaemic insult can be controlled by the length and location of the MCA segment to be electrocoagulated. Large infarcts incorporating subcortical and cortical tissue are induced by occluding the artery from a point proximal to the lenticulostriate branches to the point where the MCA crosses the inferior cerebral vein.^174^ Alternatively, occluding a distal segment of the MCA, immediately above and below the point where it crosses the inferior cerebral vein, produces a reproducible cortical lesion that incorporates the forelimb and hindlimb regions of the motor cortex, allows sensitive neurological scoring and motor deficits to be detected and is characterised by good recovery and low mortality. Shorter, distal occlusion of the artery distal to the inferior cerebral vein leads to small, variable (or no) infarcts confined to the cortex.^175^ To generate more consistent infarcts, permanent distal MCA occlusion can be combined with tandem CCA occlusion in rats.^79^ Surgeons should start with a short CCA occlusion time (e.g. 30 min) to see whether adequate lesion volumes (and/or required behavioural deficits) are obtained and only then increase CCA occlusion if necessary. Anecdotally, higher mortality is found in elderly male rather than elderly female rats with equivalent occlusion times so shorter occlusion times may be required for elderly males. Choice of the side of occlusion (left or right MCA) is subject to similar considerations to the intraluminal filament model. Another consideration is the difference in skull thickness between rodent species: mice have thinner skulls than rats, allowing relatively easier craniotomy and localization of the MCA.

In summary, the advantages of electrocoagulation models are better control over the completeness of MCAO, variability and location of ischaemic lesions (e.g. cortical, or cortical plus sub-cortical involvement) and low mortality. However, variation in the MCA anatomy may influence the size of the infarct, surgery is more invasive and challenging than for intraluminal filament MCAO, which can extend the time under general anaesthesia and there is no potential for reperfusion through the occluded artery.

## 8 Concluding remarks

Stroke models can be severe but here we present small incremental changes, which can improve the welfare of the animals used. The recommendations presented in this manuscript are recapitulated in a summary document (see supplementary information) which can be printed off as an easy reference sheet for researchers, animal welfare staff or editors. Table 5 also provides a high level summary of all the recommendations.

**Table 5.** Summary of recommendations.

Basic requirements before stroke surgery	Rodents should be group housed in appropriate housing conditions and acclimatised to their environment, cage mates, diet and monitoring procedures. Cages should include animals allocated to different groups (recommendations 1–15)
Anaesthesia and analgesia	The anaesthetic and analgesic protocols should be chosen on the basis of both welfare and scientific outcomes, and the effectiveness of pain relief should be assessed. Care should be taken to choose an anaesthetic and analgesic which are compatible with the outcomes of the experiment and so that pain is not a confound (recommendations 16–23)
Intraoperative care	Using aseptic techniques is essential and can be achieved more readily if the surgeon works with an assistant. Core temperature, cardiovascular and respiratory parameters, and depth of anaesthesia should be monitored and maintained. Intubation and artificial respiration should be considered for experimental protocols involving induction of ischaemic lesions, particularly for those lasting longer than 30 min (recommendations 24–35).
Post-operative care	Post-operative intervention, assessment points and humane endpoints should be agreed in advance with veterinary and animal care staff. Animals should be monitored frequently using a traffic light system and clinical assessment sheets; monitoring frequency should be adapted to the condition of individual animals and animals reaching a pre-defined humane endpoint should be promptly and humanely killed. Animals should be kept hydrated and provided with an appropriate post-surgical diet (recommendations 36–43).

In making these recommendations we have to acknowledge certain weaknesses in our approach. We have referred to the literature wherever possible, and where there are gaps in published information or, as is quite common, the evidence is conflicting, we have relied on the expert opinion and experience of the authors and we present here a consensus of their views. In addition, we recognise that every study has to take into account its individual constraints and objectives and therefore there is no single recipe for the best approach.

With this in mind, we emphasise the real importance of careful planning at every stage of the process, including transport, housing, acclimatisation (including novel diet), anaesthesia, analgesia, post-operative care, use of a scientific scoring system to assess wellbeing, and definition of intervention points and humane endpoints. Awareness of external developments in refinement and internal consultation with animal care staff and vets before surgery are essential.

The evidence base is currently lacking for some aspects of stroke modelling in rodents. Particularly, more research is needed to validate methods of assessing pain in stroke animals, to investigate whether and how analgesic agents interact with stroke mechanisms, and assess the impact of environmental enrichment on stroke outcomes. We emphasise the need for comprehensive reporting and disclosure of potential confounding factors in publications so that peers can interpret results, put them in context and benefit from the experiences of others.^14^

Training for researchers, students and animal technicians is of course vital and lack of training and/or resources should not be used as a reason to use lower, though legally acceptable, standards.

## Supplementary Material

Supplementary material
