# Supplementary material for: The IMPROVE Guidelines (Ischaemia Models: Procedural Refinements Of in Vivo Experiments)
Source: J Cereb Blood Flow Metab. 2017 Aug 11;37(11):3488–517. doi: 10.1177/0271678X17709185 (PMC5669349; doi:10.1177/0271678X17709185)
Supplement: Supplementary material [file JCB709185_supplementary_material.pdf]

1. Summary of all recommendations for IMPROVE (Ischaemia Models: Procedural Refinements Of *In Vivo* Experiments)

The recommendations describe opportunities to improve the *in vivo* modelling of ischaemic stroke and minimise the level of severity in the most common rodent models of cerebral ischaemia, while sustaining or improving the scientific outcomes. The aim is to provide support for researchers and animal care staff to refine their procedures and practices, and implement small incremental changes to improve the welfare of the animals used and to answer the scientific question under investigation.

TIMING		RECOMMENDATION
Basic requirements before stroke surgery	1	Rodents ordered from an outside supplier should be delivered at least seven days before the procedure to allow acclimatisation to the new environment.
	2	Animals should be acclimatised in harmonious groups before the start of the experiment. Re-housing animals in new groups should be avoided.
	3	Animals should be acclimatised to handling and should not be handled by the tail. Tunnel and cup handling should be used for mice; rats should be handled by grasping around the shoulders.
	4	Animals should be weighed daily for at least three days before surgery.
	5	Cage substrate, nesting material and shelter are basic welfare needs for rodents and should be provided. Tunnels, wheels and chewing sticks are simple, cost-effective ways to improve enrichment.
	6	Additional 'super-enrichment' should be considered carefully, as it may have neurorestorative effects. Enrichment should be reported in publications specifically.
	7	In consultation with the veterinary and animal care staff, consideration should be given to the bedding materials and any new material should be introduced prior to surgery to acclimatise the animals.
	8	After stroke, animals should be returned to the same group of animals they were with before surgery as soon as they are sufficiently recovered.
	9	The randomisation protocol should ensure that each cage contains sham-operated and stroke animals, and/or animals allocated to different treatments.
	10	Animals should have access to their post-stroke diet prior to surgery and surgery should not be undertaken until they reliably consume the diet.
	11	Rodents should not be routinely fasted before surgery, unless there is a scientific reason; any restriction should be reported specifically in publications.
	12	After any food restriction for training purposes, sufficient time should be left to re-establish normal feeding patterns before surgery.
	13	Consistency of inter-animal housing, feeding and handling practices before and after stroke should be ensured.
	14	Aged animals and those with co-morbidities should receive extra monitoring.
	15	Teeth should be checked regularly, especially if the animal is on soft food diet. Animals should be provided with chew sticks to grind teeth.
Anaesthesia and analgesia	16	The anaesthetic should be chosen on the basis of both welfare and scientific outcomes and should take account of species, strain and health status of the animals. Selection should involve the vet.
	17	Sham-operated animals should receive exactly the same anaesthesia regimen for the same duration as the test group in order to control for effects of the anaesthetic on outcomes.
	18	Local anaesthesia should be used prior to incision during surgery, particularly if other types of analgesia are not being provided, and with knowledge of local anatomy to ensure that it is applied in the appropriate area.
	19	Pain is a variable which needs to be controlled. Pain relief must be used unless there are good scientific reasons not to, supported by solid, reproducible evidence.
	20	The analgesic drug should be selected in consultation with the vet, based on the objective of the study, the specific stroke model and the type and timing of outcome measures.
	21	The animal should be assessed for level of pain post-operatively, to ensure that the analgesic regime is effective and to minimise the risk of any unnecessary medication or side-effects.
	22	All animals should either receive the same doses of analgesics to avoid pain relief being a confounder, or the experimental design and analysis should account for animals receiving different doses. This should be reported explicitly in publications.
	23	Analgesia should be given by the most reliable and least stressful route. If there is doubt about oral consumption, analgesics should be given parenterally.

TIMING		RECOMMENDATION
Intraoperative care	24	Aseptic surgical technique is essential.
	25	Antibiotics should not be used prophylactically unless there is a justified case.
	26	The surgeon should work with an assistant.
	27	Surgeons' performance should be monitored and reviewed.
	28	During general anaesthesia and in the immediate post-operative period, the animal's body temperature should be maintained by insulation or supported by a heating device, with a feedback heating system that cuts out when normal body temperature is reached.
	29	Additional care and monitoring of body temperature may be needed for obese animals.
	30	Monitoring of respiratory and cardiovascular parameters is essential for animal safety and the reproducibility of study methods.
	31	Minimum parameters to monitor are depth of anaesthesia, respiratory rate and temperature. Surgical activities should also be recorded.
	32	Pulse oximetry is recommended as SaO_2 is a good indicator of tissue oxygenation and heart rate is difficult to monitor otherwise.
	33	Invasive monitoring is useful for experiments carried out under terminal general anaesthesia or for specific cases justified by the study needs.
	34	Intubation and artificial respiration should be considered for experimental protocols involving induction of ischaemic lesions, particularly for those lasting longer than 30 minutes.
	35	Animals should be administered fluids pre-emptively to prevent dehydration during surgery.
Post-operative care	36	The experimental study plan should include details of planned post-operative intervention and assessment points. This should be devised in consultation with veterinary and animal care staff.
	37	All animals should be monitored frequently (at least 4 times a day at regular interval during the first 48h post-stroke) using a traffic light system (see table overleaf) and should be humanely killed if they reach a pre-defined humane endpoint (red status).
	38	Monitoring frequency must be increased if co-morbid animals are used and if animals are showing any clinical signs requiring intervention (amber status).
	39	Monitoring duration should be long enough to ensure eating and drinking behaviours are observed.
	40	Clinical assessment sheets should be completed each time animals are monitored – such sheets should remain with animal cages to ensure record of observations and consistency of care.
	41	Dehydration should be assessed frequently and treated post-operatively until the animal is seen to be drinking normally.
	42	Additional hydration should be provided at least for the first two days post-surgery.
	43	Animals should be provided with an appropriate post-surgical diet (e.g. wet mash). Softened food and loose pellets on the cage floor should be supplied for at least seven days post-stroke.

Reference: Percie du Sert N, Alfieri A, Allan SM, *et al.* The IMPROVE Guidelines (Ischaemia Models: Procedural Refinements Of *in Vivo* Experiments). *J Cereb Blood Flow Metab* 2017. DOI: 10.1177/0271678X17709185

NC3Rs
National Centre for the Replacement Refinement & Reduction of Animals in Research

2. Signs to monitor after experimental stroke surgery in rodent models

Clinical signs that may present following induction of cerebral ischemia have been characterized as green, amber or red, as detailed below.
The guidelines apply to adult rodents from standard, commonly used strains and may need adjustment under other circumstances (e.g. for aged, young or obese animals).

	GREEN	AMBER	RED
	Clinical signs similar to those seen in clinical stroke are expected to be present after a focal ischaemic insult as it results in brain injury and swelling. For some studies where scientific endpoints can be achieved by small infarcts, signs may be reduced in severity or absent. It is anticipated that the majority of animals will not display all of the clinical signs, and any clinical signs should start to show improvement by 48h post-stroke.	The presence of any of the signs in the amber column below requires an increase in the frequency of monitoring and appropriate intervention, such as consulting the vet or implementing some of the recommendations presented overleaf. If any of the clinical signs exceed the limits stated, the animal should automatically move to RED status.	The presence of any of the signs in the red column below requires immediate euthanasia of the animal via an approved method of humane killing (in the UK, Schedule 1 or other licensed method).
TIMING			
Anytime	• Sensorimotor deficits (e.g. forelimb weakness/hemiparesis, altered gait)<br>• Neglect of one side of the body, preference for one direction of movement<br>• Reduced food and water intake<br>• Abnormal behaviour upon handling – this may involve increased or decreased reaction to being handled compared to non-stroke animals	• Intermittent circling behaviour<br>• Absence of urine *<br>• Absence of faeces *<br>• Surgical wound complication<br>• Reduced motility, beyond what would be expected based on the severity of the ischaemic insult	• Presence of barrel rolling<br>• Presence of tonic clonic seizures<br>• Continuous laboured respiration which is beyond what is considered normal for the age/weight of animal exposed to stroke ***
First 24h post-stroke	• Lethargy and reduced motility with large strokes	• Animal not eating some normal chow or food supplement (e.g. wet mash)	• Animal not moving, unresponsive to stimulation, or in a lateral recumbent position after the normal period of time expected for recovery from general anaesthesia, given the anaesthetic used, the duration of anaesthesia and the severity of the ischaemic insult.
First 48h post-stroke	• Weight loss **<br>• Piloerection/ staring coat<br>• Discharge from the eyes and nose	• Progressive weight loss exceeding 10% of the animal´s pre-stroke weight<br>• Audible respiratory noises (rasping, wheezing), usually intermittent and normally not associated with an increased respiratory effort ***	• Weight loss of 20% despite all efforts to supplement fluid and diet. A decision not to euthanise (e.g. if animal is eating well, drinking and able to move around and explore its environment) must be independently endorsed by the vet or animal welfare officer
First week post-stroke	• Recovery of weight loss starting 4 to 5 days post stroke<br>• Disruption to nest building activity during first week post stroke – although this is strain dependent as some strains don't nest build	• Weight loss of up to 20% beyond 48h post-stroke (despite all efforts to supplement fluid and diet) but animal is eating well, drinking and able to move around and explore its environment.<br>• No recovery of weight towards pre-stroke level by 4 days post-stroke<br>• Intermittent abnormal motor activity, suggestive of seizure, during first 72h only<br>• Piloerection beyond the first 48h<br>• Reduced grooming beyond the first 48h<br>• Secretions around the nose and eyes persisting beyond first 48h<br>• No evidence of a return to near normal eating and drinking behaviour by day 4 post-stroke	• Weight loss exceeding 20% beyond 48h post-stroke despite all efforts to supplement fluid and diet. A decision not to euthanise (e.g. if animal is eating well, drinking and able to move around and explore its environment) must be independently endorsed by the vet or animal welfare officer<br>• No recovery of weight towards pre-stroke level by 7 days post-stroke. A decision not to euthanise (e.g. if the animal is eating well, drinking and exhibiting signs of normal behaviour) must be independently endorsed by the vet or animal welfare officer<br>• Intermittent abnormal motor activity, suggestive of seizure, persisting beyond first 72h<br>• Intermittent abnormal and laboured breathing (e.g. rasping) observed for two consecutive observations, or persisting beyond the first 48h ***

* These would typically be observed in singly housed animals to assess if the animal is eating and drinking sufficiently. However, group-housing has been shown to aid recovery. In group-housed animals, observing eating/drinking habits, monitoring body weight and whether the animals defecate/urinate during handling can be used to assess whether each animal is eating and drinking sufficiently.

** Weight loss after stroke is common, both in humans and in rodent models, and can be explained principally by dehydration, impaired feeding, inactivity and paralysis. However, other factors such as neuroendocrine sympathetic activation, fever and inflammation also contribute to metabolic imbalance and an increased catabolic drive leads to tissue wasting of both fat and muscle, depleting energy stores and leading to functional decline. Rodent models typically demonstrate a dramatic weight loss after stroke surgery, which normally starts recovering after 4-5 days. The amount of weight lost during that period is tightly correlated with the size of the infarct.

*** Abnormal breathing which is beyond what is considered normal for the age/weight and characteristics of animal exposed to stroke. For example, obese animals may exhibit noisier rasping respiration than non-obese animals. Intermittent wheezing (amber sign) can be seen in the first 24 hours after MCAO and is normally associated with intubation and/or long or repeated anaesthesia, most likely due to accumulation of respiratory secretions and/or minor laryngeal trauma. It should resolve within 24-36 hours. Respiratory distress (red sign) may be a result of pulmonary oedema associated with the MCAO or severe laryngeal trauma at the time of intubation.

NC3Rs
National Centre for the Replacement Refinement & Reduction of Animals in Research
